# Next-Generation Sequencing of an 88-Year-Old Specimen of the Poorly Known Species *Liagora japonica* (Nemaliales, Rhodophyta) Supports the Recognition of *Otohimella* gen. nov.

**DOI:** 10.1371/journal.pone.0158944

**Published:** 2016-07-07

**Authors:** Masahiro Suzuki, Takahiro Segawa, Hiroshi Mori, Ayumi Akiyoshi, Ryo Ootsuki, Akira Kurihara, Hidetoshi Sakayama, Taiju Kitayama, Tsuyoshi Abe, Kazuhiro Kogame, Hiroshi Kawai, Hisayoshi Nozaki

**Affiliations:** 1 Kobe University Research Center for Inland Sea, Awaji, Hyogo, Japan; 2 Center for Life Science Research, University of Yamanashi, Yamanashi, Japan; 3 National Institute of Polar Research, Midori-cho, Tachikawa-shi, Tokyo, Japan; 4 Transdisciplinary Research Integration Center, Toranomon, Minato-ku, Tokyo, Japan; 5 Department of Biological Information, Graduate School of Bioscience and Biotechnology, Tokyo Institute of Technology, Meguro-ku, Tokyo, Japan; 6 Department of Natural Sciences, Faculty of Arts and Sciences, Komazawa University, Setagaya-ku, Tokyo, Japan; 7 Faculty of Chemical and Biological Sciences, Japan Women's University, Tokyo, Japan; 8 Bioresource Sciences, Faculty of Agriculture, Kyushu University, Higashi-ku, Fukuoka, Japan; 9 Department of Biology, Graduate School of Science, Kobe University, Rokkodai, Nada-ku, Kobe, Japan; 10 Department of Botany, National Museum of Nature and Science, Tsukuba, Ibaraki, Japan; 11 The Hokkaido University Museum, Hokkaido University, Sapporo, Hokkaido, Japan; 12 Department of Biological Sciences, Faculty of Science, Hokkaido University, Sapporo, Hokkaido, Japan; 13 Department of Biological Sciences, Graduate School of Science, University of Tokyo, Hongo, Bunkyo-ku, Tokyo, Japan; Tel-Aviv University, ISRAEL

## Abstract

*Liagora japonica* is a red algal species distributed in temperate regions of Japan. This species has not been collected from its type locality on the Pacific coast of Japan since 1927 and seems to have become extinct in this area. For molecular characterization of *L*. *japonica*, we extracted DNA from the topotype material of *L*. *japonica* collected in 1927, analyzed seven genes using Illumina next-generation sequencing, and compared these data with sequences from modern samples of similar red algae collected from the Japan Sea coast of Japan. Both morphological and molecular data from modern samples and historical specimens (including the lectotype and topotype) suggest that the specimens from the Pacific and Japan Sea coasts of Japan should be treated as a single species, and that *L*. *japonica* is phylogenetically separated from the genus *Liagora*. Based on the phylogenetic results and examination of reproductive structures, we propose *Otohimella japonica* gen. et comb. nov., characterized morphologically by diffuse carposporophytes, undivided carposporangia, and involucral filaments initiated only from the cortical cell on the supporting cell.

## Introduction

Taxonomic studies are based on type specimens, which are permanently attached to taxonomic names. The type specimens of red algal seaweeds are basically specimens housed in herbaria. To determine the correct names of modern collections, type specimens have been used for comparisons, including habit and anatomy. In many cases, morphological information from type specimens is not sufficient for taxonomic studies, because many type materials are fragmented and lack reproductive structures required for comparison with modern collections. Instead of type materials, detailed morphological observations and DNA sequencing from topotypes newly collected from their type locality have been used for taxonomic studies [1, 2]. However, in some cases the coastal environment of type localities has changed and the species are missing. The most reliable method to determine the correct name of modern collections involves sequencing of type specimens and comparing them to related sequences from field-collected material [3, 4]. However, DNA fragments of less than 300 base pairs can be obtained typically [3, 4], and thus complete gene sequences required for phylogenetic analyses are not available. Many type specimens were established more than 50 years ago, and the DNA has become highly fragmented due to deterioration over time [5]; therefore, Sanger sequencing is not reliable with old DNA samples. Recently, next-generation sequencing (NGS) has been used to examine DNA sequences from old samples [6]. For red algal seaweeds, Hughey et al. [7] performed NGS to determine the complete plastid and mitochondrial genomes from 140-year-old type specimens of the bangiophycean species of *Pyropia* using the published complete organelle genome data from *Pyropia* spp. for reference mapping and sequence assembly. However, in the case of Florideophyceae, the largest group of red algae, limited genomic data are available as references in NGS, compared with bangiophycean red algae, and thus old DNA sequences have not been determined previously by NGS for molecular phylogenetic analyses.

The red algal genus *Liagora* is the largest genus of Liagoraceae and is widely distributed in warm temperate to tropical regions of the Atlantic, Indian, and Pacific Oceans [8]. Recent molecular analyses suggest that *Liagora* is polyphyletic, and the generic concept of *Liagora* sensu stricto has been revised substantially in recent years [9–11]. Although three new genera, *Macrocarpus*, *Neoizziella*, and *Titanophycus*, have since been separated from *Liagora* sensu lato [10, 12], the generic positions of some species of *Liagora* remain unresolved.

*Liagora japonica* Yamada was originally described based on plants collected from Misaki, Kanagawa Prefecture, on the Pacific side of Japan in 1903 [13]. According to our investigations of specimens housed in the Herbarium of the Graduate School of Science, Hokkaido University (SAP) and the Department of Botany, National Museum of Nature and Science, Japan (TNS), *L*. *japonica* has not been collected from its type locality since 1927, and the species has not been collected from the Pacific coast of Japan since 1960 (Figure A in S1 File and Table A in S2 File). The coast of the Miura Peninsula (which includes the type locality of *L*. *japonica*) has experienced environmental disturbances several times over the past 100 years, including vertical displacement during the 1923 Kanto Earthquake [14], landfill in Tokyo Bay in the 1950s [15], and construction of marinas for the 1964 Summer Olympics in Tokyo [16]. Several seaweeds have disappeared from the peninsula and its vicinity [15, 16], and it seems that *L*. *japonica* has become extinct at its type locality and vicinity.

Recently, we collected samples similar to *L*. *japonica* from Sado Island and Oki Island in the Japan Sea (Figure A in S1 File Tables A and B in S2 File). The collection sites are located on the side opposite the Pacific coast of Honshu, Japan, and the reproductive structures of modern samples do not correspond completely to those originally described by Yamada [13]. Therefore, it remains unclear whether modern samples collected from the Japan Sea side of Japan belong to *L*. *japonica*. To determine the identity of *L*. *japonica*, we extracted DNA from topotype material of *L*. *japonica* collected in 1927 and sequenced nuclear-encoded 28S ribosomal RNA (28S *r*RNA), plastid-encoded photosystem I P700 chlorophyll *a* apoprotein A1 (*psaA*), plastid-encoded ribulose-1,5-bisphosphate carboxylase/oxygenase (*rbc*L) genes, and the universal mitochondrial barcode marker cytochrome oxidase 1 (COI) using Illumina NGS and determined the phylogenetic position of the type material of *L*. *japonica*. We also examined herbarium specimens of lectotype and topotype as well as formalin-preserved samples used by Chihara and Yosihzaki [17] for anatomical observations. Both morphological characteristics and molecular analyses indicated that the specimens from the Pacific and Japan Sea coasts are the same species and can therefore be referred to as *L*. *japonica*. However, our molecular analyses indicated that *L*. *japonica* is incorrectly placed in *Liagora* and requires assignment to a new genus. We address the generic placement of *L*. *japonica* and propose a new genus, *Otohimella* gen. nov.

## Materials and Methods

### Ethics statement

We collected *Liagora japonica* from Sado Island and Oki Island in Japan. Collection locations and details are shown in Table B in S2 File. The collection site in Sado Island is not a protected area, while the coast of Oki Island lies within the ordinary zone of Daisen-Oki National Park. According to the National Park Act in Japan, there are no restrictions collecting marine algae from the ordinary zones of national parks. In addition, no specific permission was required for the locations on Sado Island and Oki Island.

The specimens of *L*. *japonica* housed in herbaria were subjected to morphological and molecular investigations. Official permission for this study was obtained from the Herbarium of the Graduate School of Science, Hokkaido University (SAP), and the Department of Botany, National Museum of Nature and Science, Japan (TNS).

### Morphological observations

Specimens were preserved in Silica gel for DNA extraction, or 10% formalin/seawater for anatomical observations. Voucher herbarium specimens were deposited at the Department of Botany, National Museum of Nature and Science, Japan (TNS). Lectotype and topotype specimen of *L*. *japonica*, and formalin preserved sample collected from Tsushima used for Chihara and Yoshizaki [17] were also studied. Specimens on herbarium sheets, or preserved in formalin seawater were examined under an Olympus BX50 microscope (Olympus, Japan) after the material had been rehydrated and decalcified (in some cases stained with 1% aniline blue, acidified with 1% HCl and mounted in 50% aqueous Karo^®^ syrup (Englewood Cliffs, NJ, USA) with 3% formaldehyde to prevent microbial growth. Drawings were made with the aid of a camera lucida.

### DNA extraction and sequencing procedures for modern specimens

For phylogenetic analyses, partial 28S *r*RNA, *rbc*L genes and COI were sequenced with Sanger sequencing from two modern samples (Table B in S2 File). DNA extractions and sequencing procedures were performed in S1 Text.

### DNA extraction from the historical herbarium specimens

DNA was extracted from each of the herbarium specimens, ranging in age from 88 years (collected in 1927) to 57 years (collected in 1958), using a class 100 NK System Clean Bench VSF-1600RA (NK Systems, Tokyo, Japan) to prevent microbial and human contamination. Approximately a 1 × 5 mm^2^ section of material was ruptured manually using a Handy Pestle^®^ (Toyobo, Osaka, Japan) in liquid nitrogen, and DNA was subsequently extracted using a QIAGEN^®^ Genomic-tip 20/G according to the manufacturer’s protocol (Qiagen, Valencia, CA, USA). The DNA concentration was determined using a Quant-iT dsDNA HS assay kit with a Qubit fluorometer (Life Technologies, Carlsbad, CA, USA).

Aliquot of DNA (40–80ng) from herbarium specimens were sheared to a target peak size of 500 bp using the Covaris S220 Focused-Ultrasonicator system (Covaris, Woburn, MA, USA) according to the manufacturer’s recommendations. To generate DNA sequencing libraries for high-throughput DNA sequencing, the NEBNext Ultra DNA Library Prep kit for Illumina (New England Biolabs) was used, according to the manufacturer’s instructions with the exception of performing the 10–12 cycles of PCR. The amplified library products (size range, 250–600 bp) were isolated on agarose gels and purified using the NucleoSpin Gel and PCR Clean-up kit (TaKaRa, Kyoto, Japan).

The paired-end reads were generated on the Illumina MiSeq platform using the MiSeq Reagent Kit version 2 (Illumina, San Diego, CA, USA). FASTQ files were generated using the MiSeq Reporter software version 2.3.32 (Illumina). Raw sequence reads, 10,452,985 base pairs (bp) for the 57-year-old Wakayama prefecture specimen and 9,335,303 bp for the 88-year-old Kanagawa prefecture specimen, were generated.

### Quality filtering of Illumina sequence data

We discarded the Illumina MiSeq reads that contained ambiguous nucleotides or were mapped to PhiX genomic sequences using Bowtie 2 version 2.2.3 with the default parameters [18]. Subsequently, we removed the adapter sequences from the reads, using Cutadapt version 1.2.135, and low-quality regions with a Phred-like quality score <17within the 3' end of the reads. In addition, we discarded reads that were < 20 bp in length or were associated with an average Phred-like quality score < 25. The quality filtering yielded high-quality reads: 10,057,049 bp for the 57-years-old specimen and 8,913,992 bp for the 88-year-old specimen.

### Identification of *Liagora japonica* sequences

The sequences derived from the *L*. *japonica* genomes were identified based on two different methods to avoid sequencing error. The first was a read-based method and the other a scaffold-based method.

#### Read-based method

The high-quality MiSeq reads derived from *L*. *japonica* genomes were identified as follows. (1) An in-house nucleotide sequence database consisting of the phylogenetic marker gene sequences of Nemaliales including Liagoraceae and its relatives (designated as in-house Nemaliales database) was constructed by combining previously reported sequence data (Table C in S2 File). (2) All of the high-quality reads were subjected to BLASTN 2.2.27 searches [19] against the in-house Nemaliales database with an E-value < 0.01. (3) The reads that matched the sequences in the in-house Nemaliales database were subjected to BLASTN 2.2.27 searches against the GenBank nucleotide database (September 2014) with an E-value < 0.01. (4) The reads that matched the sequences from Rhodophyta in the GenBank nucleotide database were regarded as genome fragments of *L*. *japonica*. The target read coverages of *L*. *japonica* are shown in Tables D and E in S2 File.

MiSeq reads identified as sequences belonging to nuclear genes or organelle genes of *L*. *japonica* were used for reconstruction of each gene sequence as follows. (1) In each sample, the identified reads were assembled independently for each gene using CAP3 [20]. (2) CLUSTALW was used with the default parameters to generate multiple alignments of the assembled contigs and singletons with reference gene sequences in the in-house Nemaliales database [21]. (3) Partially matched false-positive reads were removed manually by checking the multiple alignment. (4) In each sample, each gene sequence of *L*. *japonica* was reconstructed manually from the multiple alignment.

#### Scaffold-based method

The high-quality MiSeq reads of each sample were assembled using IDBA-UD version 1.1.0 with the following parameters:—mink 20—maxk 120—step 5) (Table F in S2 File) [22]. Assembled scaffolds were subjected to BLASTN 2.2.27 searches against the in-house Nemaliales database with an E-value < 0.0001. The scaffolds that matched the sequences in the in-house Nemaliales database were subjected to BLASTN 2.2.27 searches against the GenBank nucleotide database with an E-value < 0.001. The scaffolds that matched the sequences from Rhodophyta in the GenBank nucleotide database were regarded as genome fragments of *Liagora japonica*. The sequences similar to the reference nuclear gene sequences or putative organelle gene sequences were obtained from the scaffolds by (1) identifying a similar region within the reference gene sequence from the BLASTN results and (2) extracting a ±500 bp region of the BLASTN-aligned region. CLUSTAL W was used with default parameters to generate multiple alignments of the extracted sequences with reference gene sequences from the in-house Nemaliales database. Partially matched false-positive sequences were removed manually by checking the multiple alignment. In each sample, each gene sequence of *L*. *japonica* was manually reconstructed from the multiple alignment. The results of CLUSTALW and manual refinement of phylogenetic marker genes of two samples were checked, and the read coverage was calculated by Bowtie2 (version 2.2.6) mapping of the corresponding sample reads. The read alignments (BAM files) are available from the web server (http://liagora.paleogenome.jp).

### Phylogenetic analysis

We sequenced two *psaA*, four *rbc*L, four 28S *r*RNA, and four COI gene sequences from modern samples and historical herbarium specimens of *L*. *japonica* (Table B in S2 File). *PsaA*, *rbc*L, and 28S *r*RNA genes were selected to infer the phylogeny of Liagoraceae, and the COI gene was selected to assess the effectiveness of DNA barcoding. The sequence data of Liagoraceae, available from GenBank, were compiled. *PsaA* sequences for 55 taxa, *rbc*L sequences for 99 taxa, and COI sequences for 22 taxa were aligned using CLUSTAL W [21]. The 28S *r*RNA gene sequences for 51 taxa of Nemaliales were aligned using CLUSTAL W [21] and were refined based on published secondary structures of the 28S *r*RNA gene of *Palmaria palmata* (Linnaeus) Kuntze [23] using SeaView 4.1 [24]. The ambiguous regions of the alignments were removed. Samples with identical nucleotide sequences were treated as a single operational taxonomic unit (OTU). As the Liagoraceae has been resolved previously as a monophyletic group [25], four to five other families belonging to the Nemaliales were designated as outgroups for *psaA*, *rbc*L, combined *psaA* and *rbc*L, and 28S *r*RNA gene analyses. Based on the results of *psaA*, *rbc*L, combined *psaA* and *rbc*L, and 28S *r*RNA gene analyses, the genera *Cumagloia*, *Hommersandiophycus*, and *Nemalion* were designated as outgroups for COI analysis. For confirmation of the identification of 18S *r*RNA, *psaB*, and *psbA* genes derived from the historical specimens of *L*. *japonica*, phylogenetic analyses based on those genes were performed. The alignments used for the present phylogenetic analyses are available from TreeBASE at https://treebase.org/treebase-web/search/study/summary.html?id=18829 (matrix accession number S18829).

Phylogenetic analyses of the aligned sequences from each dataset, were subjected to Bayesian inference (BI) and maximum likelihood (ML) analysis. The substitution models applied to BI analyses are shown in Table G in S2 File. BI analysis was performed using MrBayes 3.2.1 [26], as described previously [27]. Four chains of Markov chain Monte Carlo (MCMC) iterations were carried out for 2,000,000 or 3,000,000 generations, keeping one tree every 500 generations. Convergence of log-likelihood and parameter values was assessed in Tracer version1.4. [28]. A burn-in sample of 5,000–7,500 trees was removed before constructing the majority rule consensus tree, and the remaining trees were used to calculate a 50% majority-rule tree and to determine the posterior probabilities (PP) of the individual branches. The Bayesian analyses are summarized in Table G in S2 File. ML analysis was performed using RAxML version7.0.4 software [29]. The GTR+I+Γ model was applied to each dataset in the analysis. Bootstrap values (BP) for ML analysis were calculated based on 1000 pseudoreplicates. The *p* distances and K2P genetic distances for each pair of liagoracean species were calculated using PAUP 4.0b10 [30].

### Nucleotide sequence accession numbers

The Sanger sequence datasets have been submitted to DDBJ under accession numbers LC066217 to LC066223, and LC066521 to LC066532 and LC093491 to LC093498, and the Illumina sequence datasets have been submitted to the DDBJ Short Read Archive under accession number DRA003813. IDBA-UD assemblies of two samples can be accessed under BCQK01000001-BCQK01275014 (suzuki-1) and BCQL01000001-BCQL01381344 (suzuki-2), respectively.

### Nomenclature acts

The electronic version of this article in Portable Document Format (PDF) in a work with an ISSN or ISBN will represent a published work according to the International Code of Nomenclature for algae, fungi, and plants, and hence the new names contained in the electronic publication of a PLOS ONE article are effectively published under that Code from the electronic edition alone, so there is no longer any need to provide printed copies.

In addition, new names contained in this work have been submitted to IPNI, from where they will be made available to the Global Names Index. The IPNI LSIDs can be resolved and the associated information viewed through any standard web browser by appending the LSID contained in this publication to the prefix http://ipni.org/. The online version of this work is archived and available from the following digital repositories: PubMed Central, LOCKSS.

## Results

### Sequences determined from historical specimens

**Fig 1:**
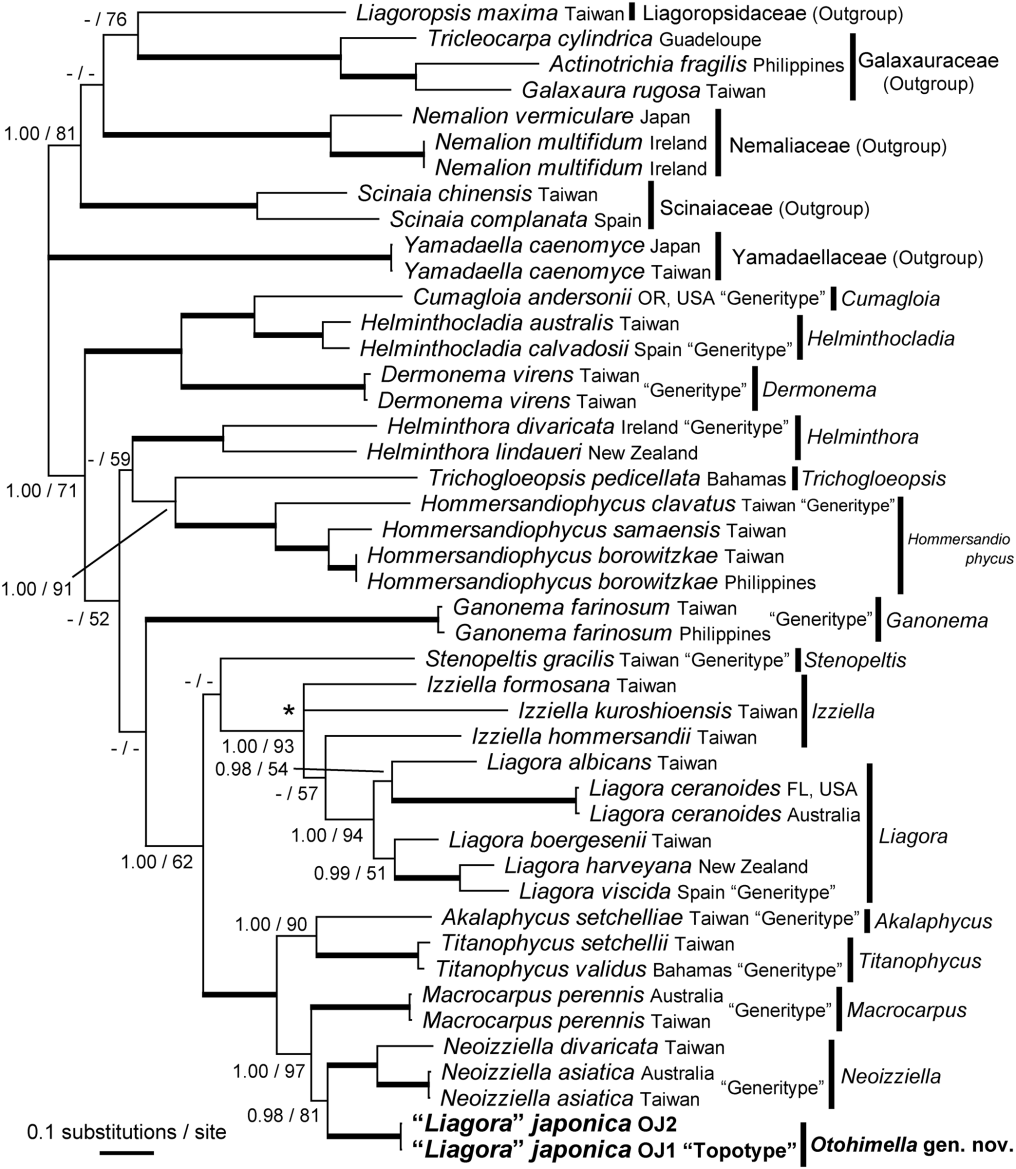
Bayesian tree based on combined *psaA* and *rbc*L gene sequences. Numbers on the branches indicate the corresponding posterior probabilities (PP, left) from Bayesian analysis and bootstrap values (BP, right) from maximum likelihood analysis. Only the PP (≥ 0.95) and BP (≥ 50%) are shown. The thick branches represent highest statistic supports (1.00 PP and 100% BP). Note that the trifurcation (asterisk) represents lack of bifurcation with 0.50 or more PP values in BI.

Based on limited publicly available sequence data from the Nemaliales, including Liagoraceae (Table C in S2 File), reliable sequences from *Liagora japonica* were determined for four genes: 28S *r*RNA, *psaA*, *rbc*L, and COI (Fig 1, Figures B-E in S1 File). Another three genes, nuclear-encoded 18S *r*RNA, putative plastid-encoded photosystem I P700 chlorophyll *a* apoprotein A2 (*psaB*), and putative plastid-encoded photosystem II core 32 kDa protein (*psbA*) genes of potentially nemalialean affinity, were obtained from the historical specimens (Figure F in S1 File, Table B in S2 File). Both the read-based and scaffold based methods, the determined sequences of the seven genes were the same. The *psaA*, *psaB*, *psbA*, *rbc*L, and COI gene sequences were complete, while only partial sequences of the 28S *r*RNA and 18S *r*RNA genes were available. The length of the 28S and 18S *r*RNA gene sequences were 82.8% and 91.5%, respectively, of the published complete 28S and 18S *r*RNA gene sequence of *P*. *palmata* [23].

### Phylogenetic markers

Analysis of the *rbc*L and COI sequences identified few polymorphisms (Figures B and E in S1 File) among populations of *L*. *japonica* from Misaki, type locality, Wakayama, Oki Island, and Sado Island. The *rbc*L gene of *L*. *japonica* showed 0–4 bp variation. The *p* distance among the species of the genus *Liagora* sensu stricto used for *rbc*L was 5.5–10.9%, whereas that among populations of *L*. *japonica* was < 0.3%. The COI gene for *L*. *japonica* showed 0–9 bp variation. The *p* distance among the species of the genus *Liagora* sensu stricto used for COI was 7.8–13.5%, whereas that among populations of *L*. *japonica* was < 1.5%. The *p* distances and K2P genetic distances among the liagoracean species used in COI analysis are shown in Tables H and I in S2 File. Both the *p* distances and K2P genetic distances generated similar results.

### Phylogenetic analyses

As *psaA*, *rbc*L, and 28S *r*RNA genes have been used for phylogenetic analyses of Liagoraceae [9–11, 25], we performed, *psaA*, *rbc*L, combined *psaA* and *rbc*L, and 28S *r*RNA gene analyses. To determine whether the third nucleotides of the codons of the *psaA* and *rbc*L genes exhibit saturation of substitutions, *p* distances among liagoracean species based on the third nucleotide of codons were compared with those of the first and second nucleotides of the same codons (Figure G in S1 File). The data indicated that these third nucleotide positions have been saturated with substitutions in liagoracean species, but not in *L*. *japonica* or four related genera: *Akalaphycus*, *Macrocarpus*, *Neoizziella*, and *Titanophycus*.

The topologies of individual *psaA*, *rbc*L, and combined *psaA* and *rbc*L trees, except the position of *L*. *japonica*, were basically similar to previous phylogenetic analyses for Liagoraceae [9–11, 25, 31, 32]. The phylogenetic trees generated by BI and ML showed the same topologies; therefore, we present only the BI tree topology (Fig 1, Figures B and C in S1 File). BI topologies of the individual *psaA* and *rbc*L trees (Figures B and C in S1 File) were similar to the combined *psaA* and *rbc*L data (Fig 1), except for the position of *Neoizziella*, but with weaker statistical support. In the individual *rbc*L tree, *L*. *japonica* and *Macrocarpus* were sisters to each other, with low support (< 0.95 PP and 59% BP), while in the individual *psaA* and combined *psaA* and *rbc*L trees, *L*. *japonica* and *Neoizziella* were sisters to each other, with moderate to high support (0.98–0.99 PP and 79–81% BP). In the combined *psaA* and *rbc*L tree, the monophyly of each genus belonging to Liagoraceae was highly supported (1.00 PP and > 95% BP), excluding *Izziella*. *Liagora japonica*, *Macrocarpus*, and *Neoizziella* formed a monophyletic clade with full support (1.00 PP and 100% BP), whereas *L*. *japonica* was clearly separated from *Liagora* sensu stricto.

The topology of the 28S *r*RNA gene tree, except the position of *L*. *japonica* was similar to previous phylogenetic analyses for Liagoraceae [10, 12, 33]. In the tree based on the 28S *r*RNA gene, *Liagora japonica* was separated from *Liagora* sensu stricto; however, the boundaries of the genera belonging to Liagoraceae were not resolved (Figure D in S1 File).

### Morphological observations of modern samples

**Fig 2:**
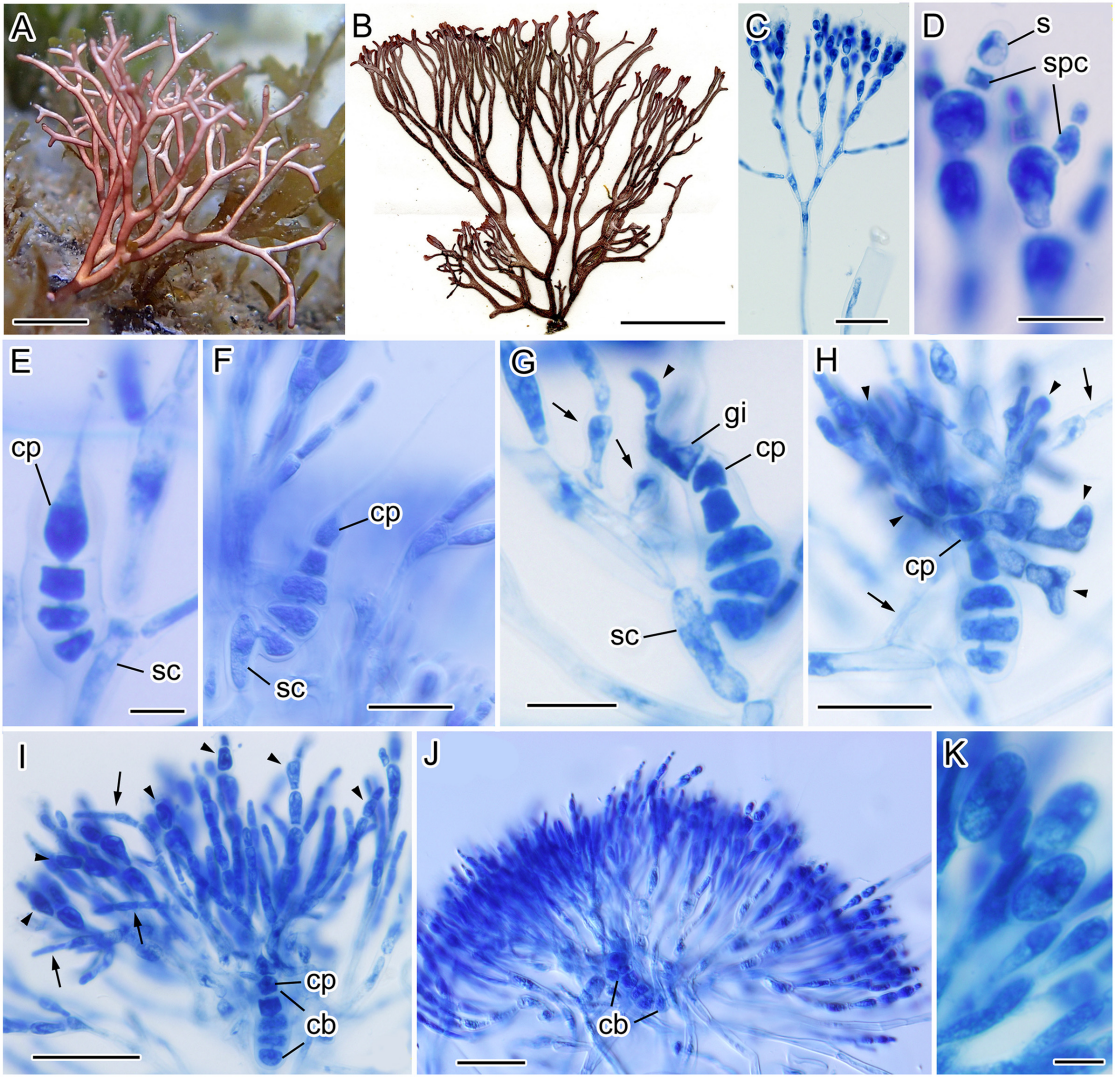
Morphological examination of modern samples of *Liagora japonica* Yamada. A: Habit (TNS-AL 195934). Scale bar = 1.0 cm. B: Herbarium specimen (TNS-AL 185628). Scale bar = 2.0 cm. C: Assimilatory filaments (TNS-AL 182118). Scale bar = 50 μm. D: Spermatangia (s) cut off from spermatangial parental cell (spc) (TNS-AL 190026). Scale bar = 10 μm. E: 4-celled carpogonial branch (TNS-AL 190026). cp = carpogonium. sc = supporting cell. Scale bar = 10 μm. F: 5-celled carpogonial branch (TNS-AL 182118). Scale bar = 30 μm. G: An early post-fertilization stage showing gonimoblast initial (gi), gonimoblast cells (arrowhead), and involucral filaments (arrows) (TNS-AL 182118). Scale bar = 20 μm. H: A later post-fertilization stage showing developing gonimoblast cells (arrowheads) and involucral filament (arrows) (TNS-AL 182118). Scale bar = 30 μm. I: Young carposporophyte showing the growth of the gonimoblast cells (arrowheads) and involucral filaments (arrows) (TNS-AL 182118). Note that the cells of carpogonial branch (cb) are not fused. Scale bar = 50 μm. J: Mature carposporophyte showing diffuse carposporophyte (TNS-AL 182118). Note that the cells of carpogonial branch remain distinct. Scale bar = 50 μm. K: Carposporangia (TNS-AL 190026). Note that carposporangia are not divided. Scale bar = 10 μm.

Thalli were found on rocks at a depth of approximately 1–3 m (Fig 2A). The thalli were erect, 4–7 cm in height, and composed of 2–3 terete axes branched subdichotomously to 5–7 orders, arising from a small discoid holdfast, loosely calcified, pinkish or reddish brown in color (Fig 2A and 2B).

The thalli were multiaxial and composed of assimilatory filaments and medullary filaments (Fig 2C). The assimilatory filaments were subdichotomously branched 3–4 times. The upper parts of the assimilatory filaments were composed of ellipsoidal cells, which were 10–18 μm long and 5–8 μm wide. The lower parts were composed of elongated cells, which were 30–60 μm long and 4–6 μm wide.

**Fig 3:**
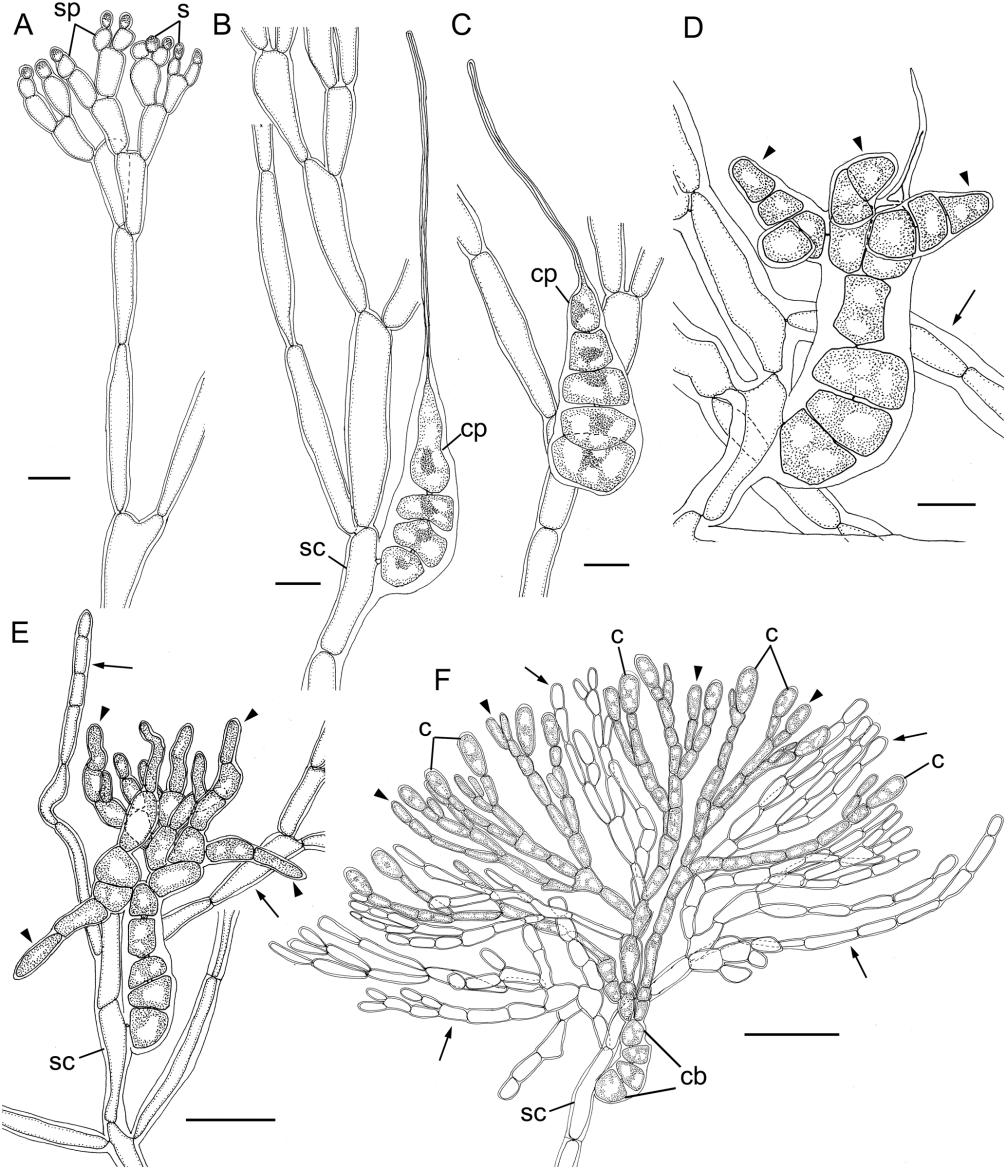
Reproductive structures of modern samples of *Liagora japonica* Yamada. A: Spermatangia (s) cut off from spermatangial parental cell (spc) (TNS-AL 190026). Scale bar = 10 μm. B: 4-celled carpogonial branch (TNS-AL 190026). cp = carpogonium. sc = supporting cell. Scale bar = 10 μm. C: 5-celled carpogonial branch (TNS-AL 190026). Scale bar = 10 μm. D: An early post-fertilization stage showing gonimoblast cells (arrowhead) and involucral filaments (arrows) (TNS-AL 190026). Scale bar = 10 μm. E: A later post-fertilization stage showing developing gonimoblast cells (arrowheads) and involucral filaments (arrows) (TNS-AL 182118). Scale bar = 30 μm. F: Mature carposporophyte showing diffuse carposporophyte (TNS-AL 182118). Scale bar = 50 μm.

Gametophytes were monoecious. Spermatangial parent cells derived from apical cells and spermatangia were cut off terminally (Figs 2D and 3A). One or, rarely, two spermatangia were cut off from each spermatangial parental cell.

Carpogonial branches were curved slightly, 4- or 5-celled, and borne on the middle part of assimilatory filaments (Figs 2E and 2F, 3B and 3C). After presumed fertilization, the carpogonium divided transversely to produce a gonimoblast initial, after which the gonimoblast initial cut off primary gonimoblast cells obliquely (Fig 2G). Meanwhile, the involucral filaments were initiated mostly from cortical cells on the supporting cell (Figs 2G, 3D and 3E). Gonimoblast initials cut off second and third gonimoblast cells, and the gonimoblast developed radially (Figs 2H, 3D and 3E). At an early stage of gonimoblast development, growth of the involucral filaments and gonimoblast cells became dominant (Fig 2I). At maturity, the gonimoblast cells were embedded in and intermingled with the involucral filaments, and carposporophytes were diffuse (Figs 2J and 3F). The cells of the carpogonial branch were not fused through carposporophyte development (Figs 2G–2J and 3D–3F). Carposporangia were elliptical to oblong, 10–15 μm long and 5–6 μm wide (Fig 2K).

### Morphological observations of historical materials

**Fig 4:**
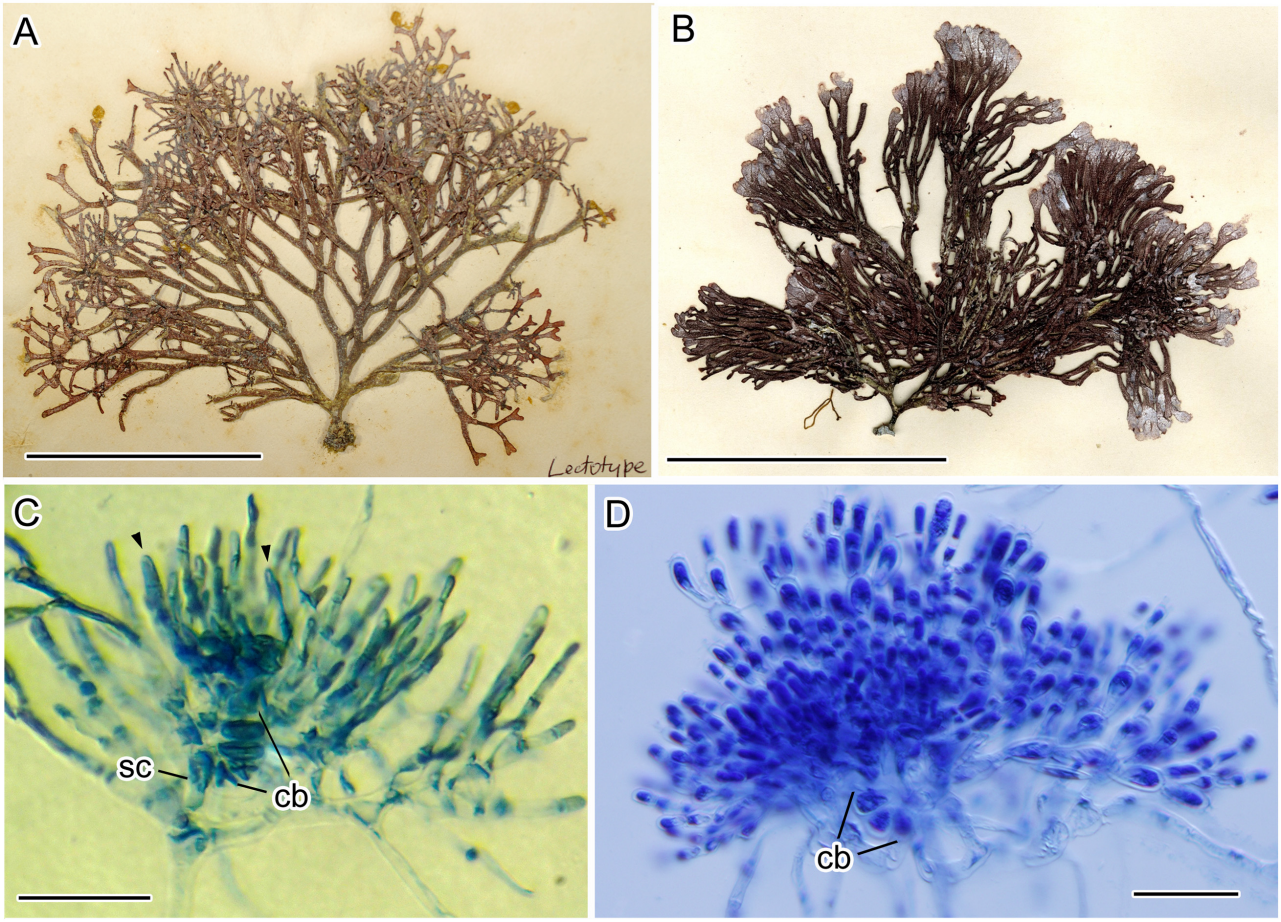
Morphological examination of historical materials of *Liagora japonica* Yamada. A: Lectotype specimen. Scale bar = 2.0 cm. B: Herbarium specimen used in Chihara & Yoshizaki (1972) (TNS-AL 047542). Scale bar = 2.0 cm. C: A later post-fertilization stage showing developing gonimoblast cells (arrowheads) (Lectotype). Scale bar = 30 μm. D: Mature carposporophyte showing diffuse carposporophyte (TNS-AL 047542). Scale bar = 50 μm.

Based on habit and vegetative anatomies, the lectotype and topotype specimens examined corresponded to the descriptions of Yamada [13], while samples collected from Tsushima fit those of Chihara and Yoshizaki [17]. Thalli were erect and bushy, consisting of 3–5 main axes, subdichotomously branched, with 3–9 orders of branching, 7–18 cm in height, arising from a discoid holdfast with moderately calcified branches (Fig 4A and 4B). After fertilization, cells of the carpogonial branch were not fused. At an early stage of gonimoblast development, gonimoblast cells were loosely elongated, and involucral filaments intermingled with gonimoblast cells (Fig 4C). Mature carposporophytes were not observed in the lectotype or topotype, while those of Tsushima were diffuse (Fig 4D).

## Discussion

The *rbc*L and COI have been used for species delineation of Nemaliales, including Liagoraceae [9, 11, 34]. Analyses of *rbc*L and COI sequences suggest that modern and historical specimens collected from four localities of both the Japan Sea and Pacific sides of Japan, including topotypes, are very closely related. The typical intraspecific divergence of *rbc*L of the Liagoraceae based on *p* distances is < 0.4% [31], while that of COI of the Nemaliales is < 1.0% [34]. *Liagora japonica* showed < 0.3% intraspecific divergence of *rbc*L, which was within the range of that of Liagoraceae [31], while the intraspecific divergence of COI was < 1.5%, which was higher than typical intraspecific divergence of the Nemaliales [34]. The typical minimum interspecific divergence of COI of the Liagoraceae is 5.6% [32], and thus the intraspecific divergence of *L*. *japonica* is much lower than the interspecific divergence. Thus, all specimens should be identified as a single species: *L*. *japonica*.

However, the structures of carposporophytes from modern samples differed from those originally described by Yamada [13]. Yamada [13] presented a drawing of compact carposporophytes, whereas carposporophytes of the modern samples were diffuse. Furthermore, Chihara and Yoshizaki [17] observed *L*. *japonica* collected from Tsushima, Japan, and also presented a drawing of compact carposporophytes, similar to Yamada [13]. However, the present examination of lectotype and topotype specimens, as well as specimens used by Chihara and Yoshizaki [17], showed elongated gonimoblast cells and involucral filaments intermingled with gonimoblast cells, similar to the carposporophyte development of diffuse carposporophytes (Table 1). The habit and vegetative structures of the modern samples collected from the Japan Sea correspond to the original description of Yamada [13]. Thus, Yamada [13] and Chihara and Yoshizaki [17] did not recognize diffuse carposporophytes, because those of the genus *Liagora* sensu lato were not recognized until the 1990s [35, 36].

**Table 1:** Morphological comparison among the description of Yamada [13] and Chihara and Yoshizaki [17], type specimens, and related samples with *Liagora japonica*.

	Yamada [13]	Chihara and Yoshizaki [17]	OJ5	OJ1	OJ3	OJ4	OJ10
Specimen	-	-	Lectotype	Topotype SAP88755	TNS-AL 182118	TNS-AL 195934	TNS-AL 029955^a^
Locality	Misaki	Tsushima	Misaki	Misaki	Sado Island	Oki Island	Tsushima
Accession No. of *rbc*L gene sequence	-	-	-	LC066217	LC066219	LC066220	-
Fusion of the cells of carpogonial branch	Not observed	Absent	Absent	Absent	Absent	Absent	Absent
Development of gonimoblast cells	Not observed	Not observed	Elongated	Elongated	Elongated	Elongated	Elongated
Development of involcural filamnets			Intermingled with gonimoblast cells	Intermingled with gonimoblast cells	Intermingled with gonimoblast cells	Intermingled with gonimoblast cells	Intermingled with gonimoblast cells
Shape of carposporophyte	Rather loose^b^	Globular aggregation	Not observed	Not observed	Diffuse	Diffuse	Diffuse

^a^Voucher specimen of Chihara & Yoshizaki [17].

^b^Yamada’s [13] Fig.10D shows compact carposporophyte.

Liagoracean genera have been recognized based on the characteristics of the structures of carposporophyte development including (1) carpogonial branches and whether they form a fusion cell; (2) the origin of the involucral filaments, a specially produced sterile cell; (3) association of the gonimoblast with paraphyses, a specially produced assimilatory filament around the carposporophyte; (4) compact or diffuse carposporophytes; and (5) division of carposporangia [9–12, 34]. Lin et al. [9–11] suggested that the genus *Liagora* is polyphyletic and should be separated into several genera based on the characteristics of carposporophyte development. Recent taxonomic studies of *Liagora* sensu stricto, including the type species *L*. *viscida* (Forsskål) C. Agardh, suggested that *Liagora* sensu stricto is characterized by fused carpogonial branching and diffuse carposporophyte [9, 37]. Three new genera, *Macrocarpus*, *Neoizziella*, and *Yoshizakia* including species with unfused carpogonial branches and diffuse carposporophytes, were separated from *Liagora* sensu stricto [9, 31]. The other liagoracean genera, *Akalaphycus*, *Patenocarpus*, and *Stenopeltis*, also produce diffuse carposporophytes; however, they produce paraphyses [33, 38]. *Liagora japonica* produces unfused carpogonial branches and diffuse carposporophytes without paraphyses, which are similar to those of *Macrocarpus*, *Neoizziella*, and *Yoshizakia*. *Macrocarpus* is characterized by divided carposporangia, while *Neoizziella* is characterized by the position of involucral filaments, which are produced from cortical cells in the vicinity of the supporting cell [9]. *Liagora japonica* differs from *Macrocarpus* and *Neoizziella* by undivided carposporangia and involucral filaments produced from the cortical cell on the supporting cell (Table 2). The critical features of carposporophyte development of *L*. *japonica* are most similar to those of *Yoshizakia*. *Yoshizakia* is characterized by involucral filaments, which are not intermingled with gonimoblast filaments. *Liagora japonica*, however, has involucral filaments that intermingle with gonimoblast filaments. Individual *psaA* and *rbc*L, and combined *psaA* and *rbc*L analyses suggested that *L*. *japonica*, *Macrocarpus*, and *Neoizziella* form a monophyletic clade separated from *Liagora* sensu stricto. However, *L*. *japonica* was not included in the clade of *Macrocarpus* or *Neoizziella*. Unfortunately, *psaA* sequence data for the species of *Yoshizakia* are not yet available, but individual *rbc*L analyses suggest that *L*. *japonica* is clearly separated from *Yoshizakia*. Both morphological and molecular results suggest that *L*. *japonica* is a distinct genus in the Liagoraceae, and we propose a new genus, *Otohimella* Mas. Suzuki, to accommodate this species.

**Table 2:** Comparisons of features distinguishing among *Liagora japonica*, *Liagora* sensu stricto, and the genera with diffuse carposporophyte belonging to Liagoraceae.

	*Liagora japonica*	*Liagora* sensu stricto	*Akalaphycus*	*Macrocarpus*	*Neoizziella*	*Patenocarpus*	*Stenopeltis*	*Yoshizakia*
Type species	-	*L*. *viscida*	*A*. *setchelliae*	*M*. *perennis*	*N*. *asiatica*	*P*. *paraphysiferus*	*S*. *gracilis*	*Y*. *indopacifica*
Association of gonimoblast with paraphyses	Absent	Absent	Present	Absent	Absent	Present	Present	Absent
Fusion of cells of carpogonial branch after fertilisation	Absent	Present	Absent	Absent	Absent	Absent	Absent	Absent
Involucral filament	Present	Present	Absent	Present	Present	Present	Absent	Present
Origin of involucral filaments	Cortical cell on the supporting cell	Cortical cells in the vicinity of the supporting cell or cortical cell on the supporting cell	N.A.	Cortical cell on the supporting cell	Cortical cells in the vicinity of the supporting cell	Cortical cells in the vicinity of the supporting cell	N.A.	Cortical cell on the supporting cell
The gonimoblast intermingled with the involucral filaments	Present	Present	N.A.	Present	Present	Present	N.A.	Absent
Division of carposporangia	Absent	Absent or present	Absent	Present	Absent	Absent	Absent	Absent
References	This study	[10, 31, 37]	[34]	[9]	[9]	[39]	[33]	[31]

### Taxonomic Treatment

*Otohimella* Mas. Suzuki gen. nov.

Description: Thalli are moderately calcified, and 5 – 18 cm in height, arising from a discoid holdfast with a short stipe. Thalli are subdichotomously branched to 5 or 6 orders. The cells of assimilatory filaments are ovoid to ellipsoidal and borne on colorless medullary filaments. Gametophytes are monoecious. Spermatangia are produced terminally on spermatangial parent cells. Carpogonial branches are slightly curved, and 4- or 5-celled. After fertilization, cells of the carpogonial branch are not fused through carposporophyte development. The involucral filaments are initiated mostly from the cortical cell on the supporting cell and eventually intermingled with the gonimoblast filaments. Mature carposporophytes are diffuse. Carposporangia are formed at the distal ends of gonimoblast filaments and are not divided.

Generitype: *Otohimella japonica* (Yamada) Mas. Suzuki, T. Segawa, Hi. Mori et Nozaki comb. nov.

Basionym of *O*. *japonica*: *Liagora japonica* Yamada. Sci. Pap. Inst. Algol. Res., Fac. Sci., Hokkaido Imp. Univ. 2: 16–17 Fig 9 and 10, Pl. IV (1938).

Etymology: Named for Otohime, a sea goddess in Japanese mythology.

## Conclusion

NGS can be used to determine DNA sequences from historical herbarium specimens containing highly fragmented DNA molecules. The liagoracean species are non-model organisms; thus, there are no useful complete genome sequences in the GenBank database for reference mapping and sequence assembly of NGS. However, we were able to determine seven gene sequences from historical herbarium specimens of *L*. *japonica* using NGS with limited reference sequence data. Four of the seven genes can be used for phylogenetic analyses and species identification. This study showed that sequencing ofhistorical specimens using NGS is a powerful tool for systematics and identification of not only model, but also non-model, organisms.

We addressed the identity of *L*. *japonica* based on both morphological and molecular data, including those of the lectotype and topotype specimens. The species had been considered extinct on the Pacific Ocean side of Japan. However, we showed that *L*. *japonica* survives on the Japan Sea side of Japan. Further, we propose *Otohimella japonica* gen. et comb. nov. based on this species.

## Supporting Information

S1 File **Figure A. Geographical distribution of *Liagora japonica* based on the herbarium specimen deposited in SAP and TNS.** Detail of collection data is shown in Tables A and B in S2 File. **Figure B. Bayesian tree based on *psaA* gene sequences.** Numbers on the branches indicate the corresponding posterior probabilities (PP, left) from Bayesian analysis and bootstrap values (BP, right) from maximum likelihood analysis. Only the PP (≥ 0.95) and BP (≥ 50%) are shown. The thick branches represent highest statistic supports (1.00 PP and 100% BP). **Figure C. Bayesian tree based on *rbc*L gene sequences.** Numbers on the branches indicate the corresponding posterior probabilities (PP, left) from Bayesian analysis and bootstrap values (BP, right) from maximum likelihood analysis. Only the PP (≥ 0.95) and BP (≥ 50%) are shown. The thick branches represent highest statistic supports (1.00 PP and 100% BP). Note that the trifurcation (asterisk) represents lack of bifurcation with 0.50 or more PP values in BI. **Figure D. Bayesian tree based on 28S *r*RNA gene sequences.** Numbers on the branches indicate the corresponding posterior probabilities (PP, left) from Bayesian analysis and bootstrap values (BP, right) from maximum likelihood analysis. Only the PP (≥ 0.95) and BP (≥ 50%) are shown. The thick branches represent highest statistic supports (1.00 PP and 100% BP). Note that the trifurcation (asterisk) represents lack of bifurcation with 0.50 or more PP values in BI. **Figure E. Bayesian tree based on COI gene sequences.** Numbers on the branches indicate the corresponding posterior probabilities (PP, left) from Bayesian analysis and bootstrap values (BP, right) from maximum likelihood analysis. Only the PP (≥ 0.95) and BP (≥ 50%) are shown. The thick branches represent highest statistic supports (1.00 PP and 100% BP). Note that the trifurcation (asterisk) represents lack of bifurcation with 0.50 or more PP values in BI. **Figure F. Bayesian tree based on 18S *r*RNA (A), *psaB* (B), and *psbA* (C) gene sequences.** Numbers on the branches indicate the corresponding posterior probabilities (PP, left) from Bayesian analysis and bootstrap values (BP, right) from maximum likelihood analysis. Only the PP (≥ 0.95) and BP (≥ 50%) are shown. The thick branches represent highest statistic supports (1.00 PP and 100% BP). Note that the trifurcation (asterisk) represents lack of bifurcation with 0.50 or more PP values in BI. **Figure G. Comparson of *p* distances among the liagoracean species based on the first and second nucleotides of codons and based on the third nucleotide of codons in the combined *psaA* and *rbc*L dataset used for the present phylogenetic analyses (Fig 1).** Red diamonds indicate *p* distances among “*Liagora*” *japonica*, *Akalaphycus*, *Macrocarpus*, *Neoizziella*, and *Titanophycus*.
(PDF)

S2 File Table A. Collection and herbarium information for specimens of *Liagora japonica* used in the morphological analyses that have no molecular data. Table B. Collection locations and details, and GenBank accession numbers of samples used in the *psaA*, *psaB*, *psbA*, *rbc*L, COI, 18S *r*RNA, and 28S *r*RNA genes analyses. Table C. GenBank accession numbers of species used in the identification of *Liagora japonica* sequences from old herbarium specimens. Table D. Target read coverage of *Liagora japonica* (sample ID: suzuki-2; sample No.: OJ1). Table E. Target read coverage of *Liagora japonica* (sample ID: suzuki-1; sample No.: OJ2). Table F. De novo assembly statistics. Table G. Summary for the Bayesian analyses on the basis of *psaA*, *psaB*, *psbA*, *rbc*L, 18S *r*RNA, and 28S *r*RNA datasets. Table H. Matrix of *p* distances of the liagoracean species used in the COI analysis. Table I. Matrix of Kimura 2-parameter (K2P) genetic distances of the liagoracean species used in the COI analysis.
(DOC)

S1 Text The DNA extraction and sequencing procedures for modern specimens. (DOCX)
