# Supplementary material for: Next-Generation Sequencing of an 88-Year-Old Specimen of the Poorly Known Species Liagora japonica (Nemaliales, Rhodophyta) Supports the Recognition of Otohimella gen. nov
Source: PLoS One. 2016 Jul 7;11(7):e0158944. doi: 10.1371/journal.pone.0158944 (PMC4936710; doi:10.1371/journal.pone.0158944)
Supplement: S1 File — Figure A. Geographical distribution of Liagora japonica based on the herbarium specimen deposited in SAP and TNS. Detail of collection data is shown in Tables A and B in S2 File. Figure B. Bayesian tree based on psaA gene sequences. Numbers on the branches indicate the corresponding posterior probabilities (PP, left) from Bayesian analysis and bootstrap values (BP, right) from maximum likelihood analysis. Only the PP (≥ 0.95) and BP (≥ 50%) are shown. The thick branches represent highest statistic supports (1.00 PP and 100% BP). Figure C. Bayesian tree based on rbcL gene sequences. Numbers on the branches indicate the corresponding posterior probabilities (PP, left) from Bayesian analysis and bootstrap values (BP, right) from maximum likelihood analysis. Only the PP (≥ 0.95) and BP (≥ 50%) are shown. The thick branches represent highest statistic supports (1.00 PP and 100% BP). Note that the trifurcation (asterisk) represents lack of bifurcation with 0.50 or more PP values in BI. Figure D. Bayesian tree based on 28S rRNA gene sequences. Numbers on the branches indicate the corresponding posterior probabilities (PP, left) from Bayesian analysis and bootstrap values (BP, right) from maximum likelihood analysis. Only the PP (≥ 0.95) and BP (≥ 50%) are shown. The thick branches represent highest statistic supports (1.00 PP and 100% BP). Note that the trifurcation (asterisk) represents lack of bifurcation with 0.50 or more PP values in BI. Figure E. Bayesian tree based on COI gene sequences. Numbers on the branches indicate the corresponding posterior probabilities (PP, left) from Bayesian analysis and bootstrap values (BP, right) from maximum likelihood analysis. Only the PP (≥ 0.95) and BP (≥ 50%) are shown. The thick branches represent highest statistic supports (1.00 PP and 100% BP). Note that the trifurcation (asterisk) represents lack of bifurcation with 0.50 or more PP values in BI. Figure F. Bayesian tree based on 18S rRNA (A), psaB (B), and psbA (C) gene sequenc [file pone.0158944.s001.pdf]

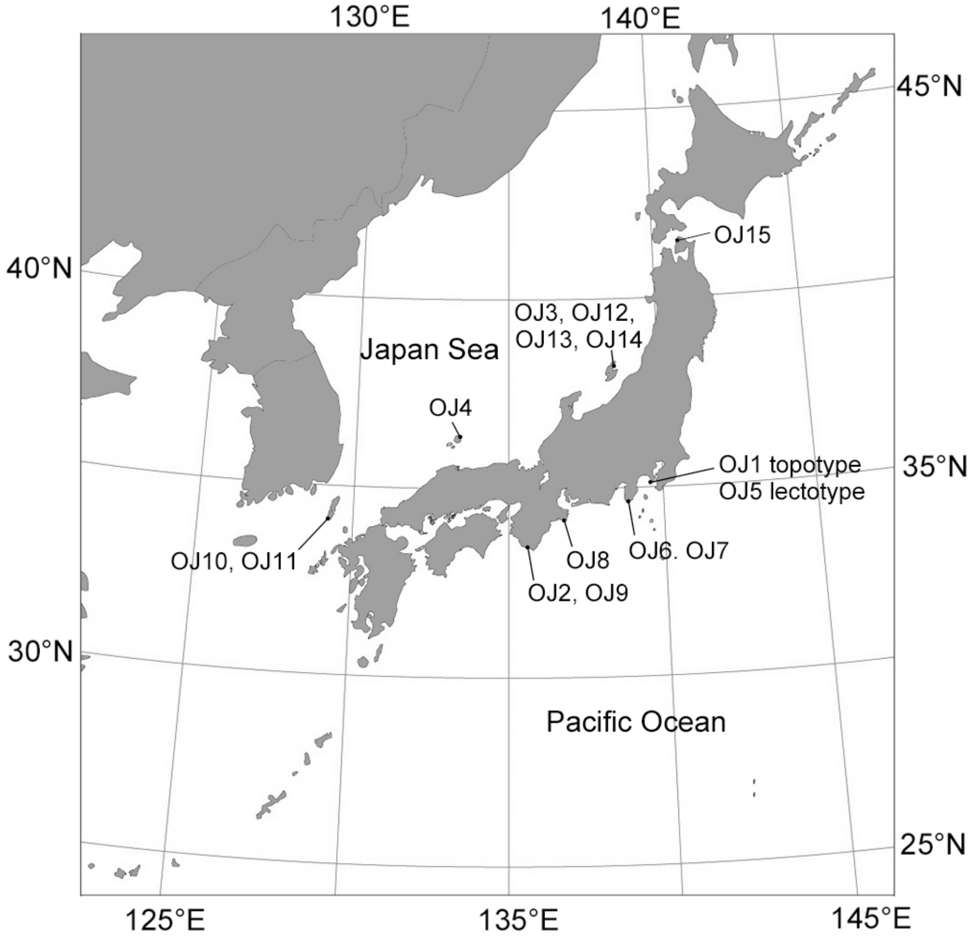

Figure A. Geographical distribution of *Liagora japonica* based on the herbarium specimen housed in SAP and TNS. Detail of collection data is shown in S1 and S2 Tables.

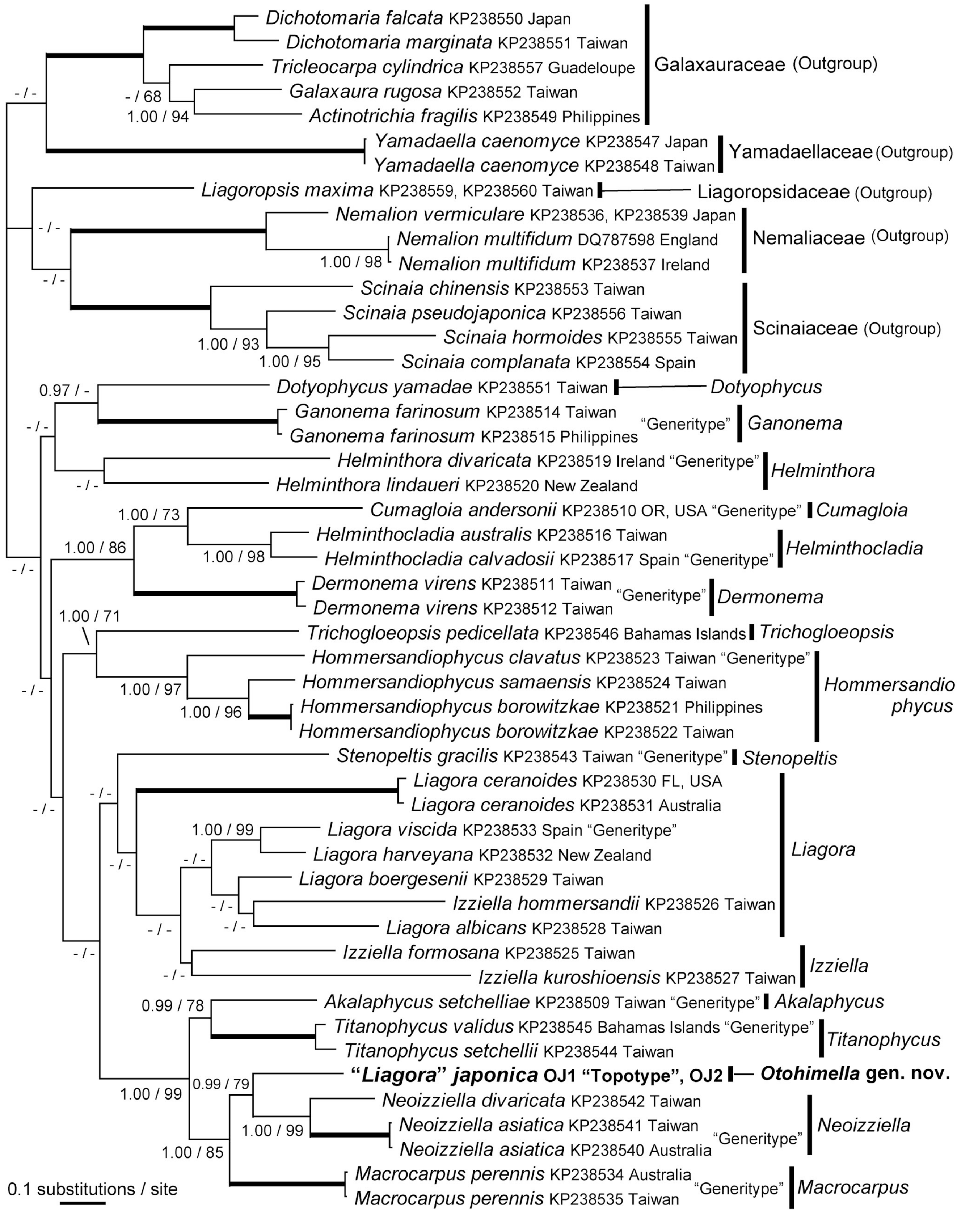

Figure B. Bayesian tree based on *psaA* gene sequences. Numbers on the branches indicate the corresponding posterior probabilities (PP, left) from Bayesian analysis and bootstrap values (BP, right) from maximum likelihood analysis. Only the PP (≥ 0.95) and BP (≥ 50%) are shown. The thick branches represent highest statistic supports (1.00 PP and 100% BP).

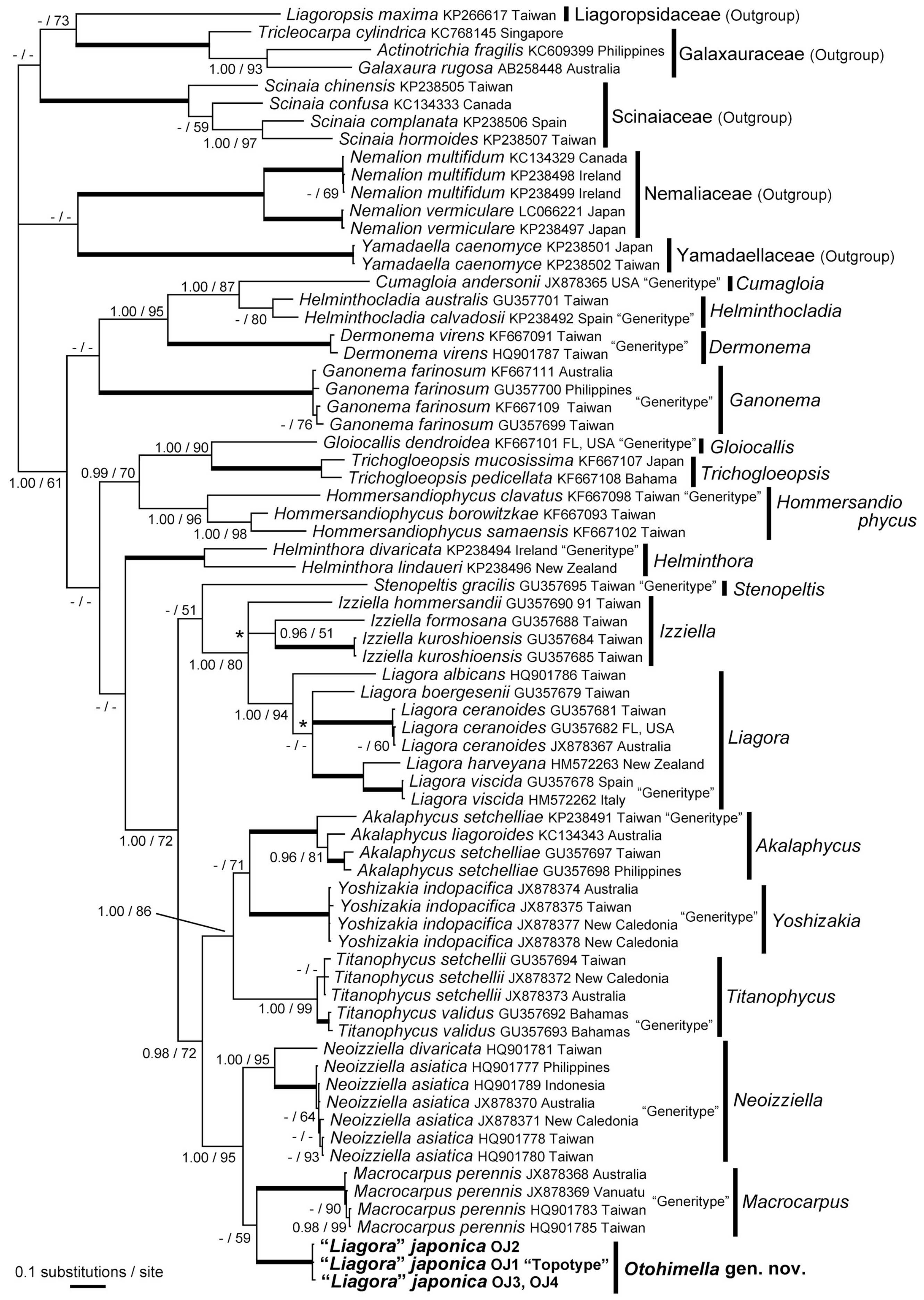

Figure C. Bayesian tree based on *rbc*L gene sequences. Numbers on the branches indicate the corresponding posterior probabilities (PP, left) from Bayesian analysis and bootstrap values (BP, right) from maximum likelihood analysis. Only the PP (≥ 0.95) and BP (≥ 50%) are shown. The thick branches represent highest statistic supports (1.00 PP and 100% BP). Note that the trifurcation (asterisk) represents lack of bifurcation with 0.50 or more PP values in BI.

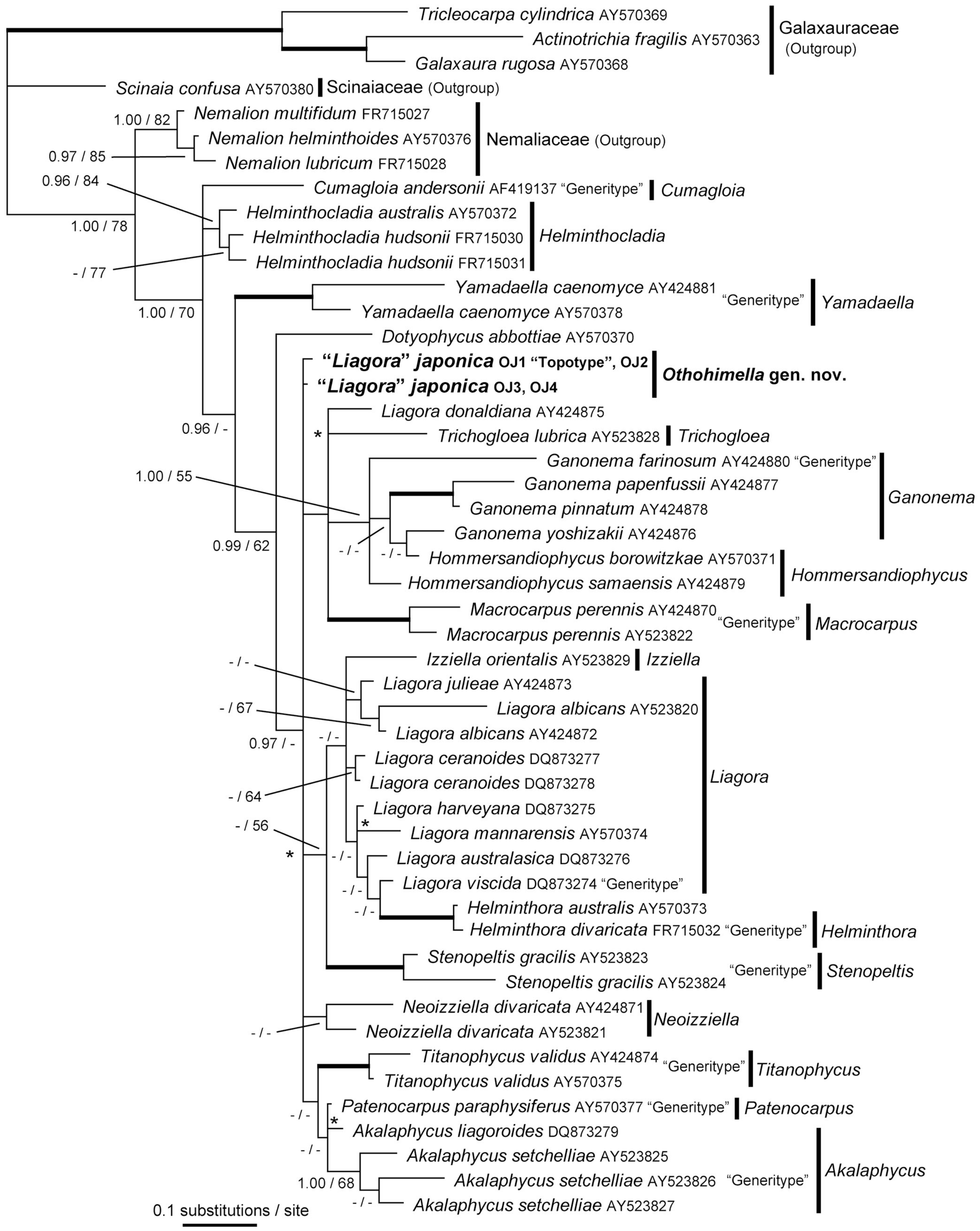

Figure D. Bayesian tree based on 28S *r*RNA gene sequences. Numbers on the branches indicate the corresponding posterior probabilities (PP, left) from Bayesian analysis and bootstrap values (BP, right) from maximum likelihood analysis. Only the PP (≥ 0.95) and BP (≥ 50%) are shown. The thick branches represent highest statistic supports (1.00 PP and 100% BP). Note that the trifurcation (asterisk) represents lack of bifurcation with 0.50 or more PP values in BI.

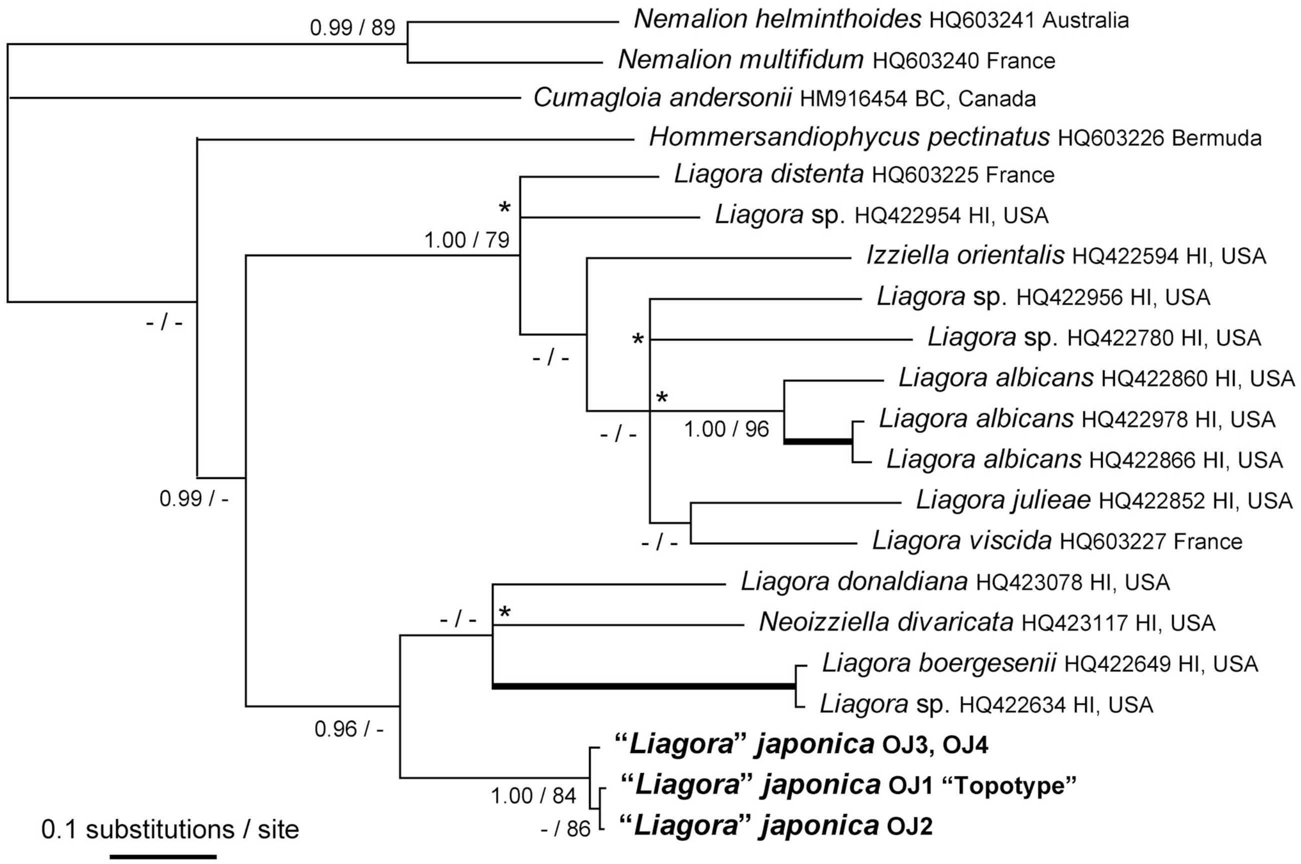

Figure E. Bayesian tree based on COI gene sequences. Numbers on the branches indicate the corresponding posterior probabilities (PP, left) from Bayesian analysis and bootstrap values (BP, right) from maximum likelihood analysis. Only the PP (≥ 0.95) and BP (≥ 50%) are shown. The thick branches represent highest statistic supports (1.00 PP and 100% BP). Note that the trifurcation (asterisk) represents lack of bifurcation with 0.50 or more PP values in BI.

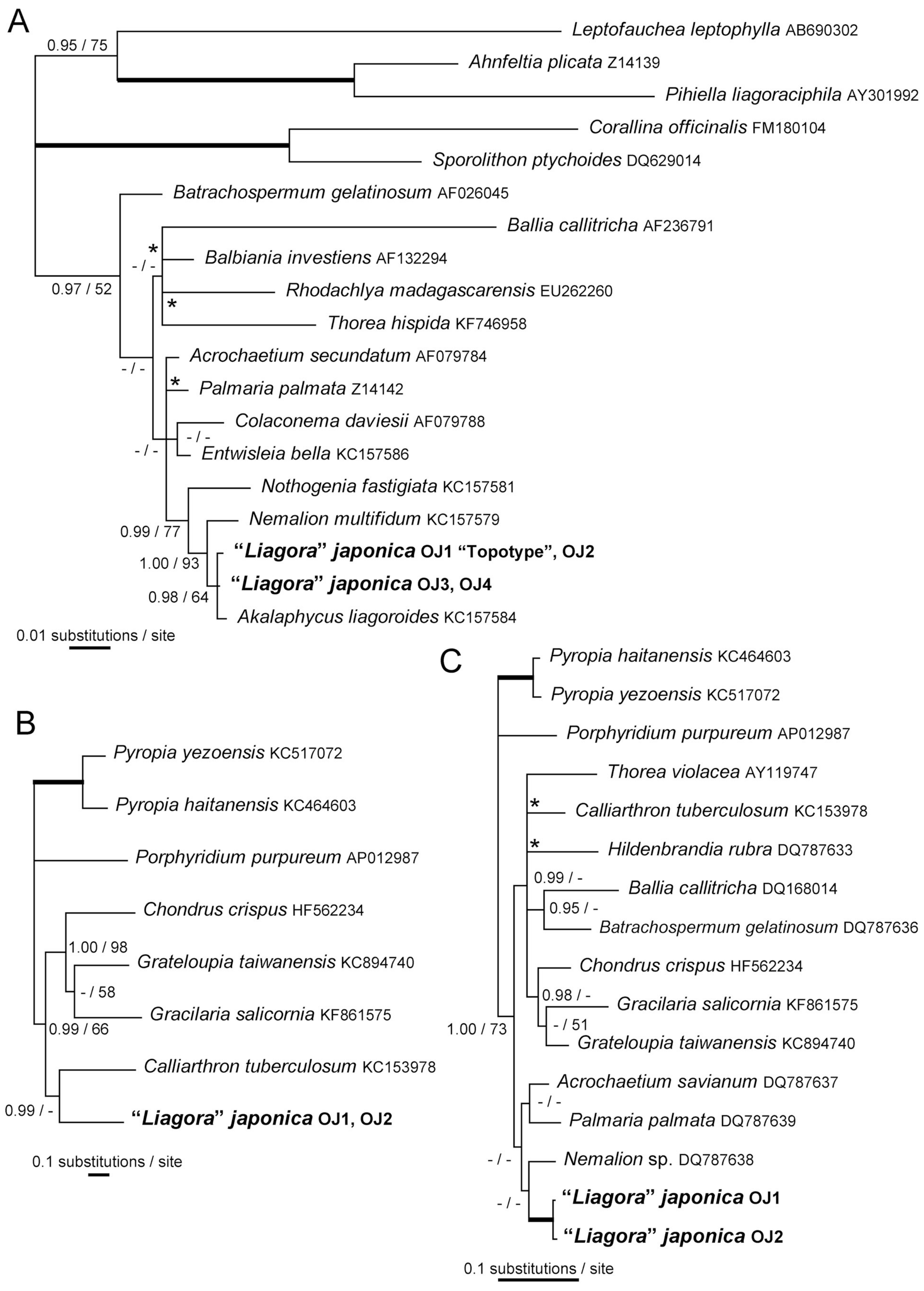

Figure F. Bayesian tree based on 18S *r*RNA (A), *psaB* (B), and *psbA* (C) gene sequences. Numbers on the branches indicate the corresponding posterior probabilities (PP, left) from Bayesian analysis and bootstrap values (BP, right) from maximum likelihood analysis. Only the PP (≥ 0.95) and BP (≥ 50%) are shown. The thick branches represent highest statistic supports (1.00 PP and 100% BP). Note that the trifurcation (asterisk) represents lack of bifurcation with 0.50 or more PP values in BI.

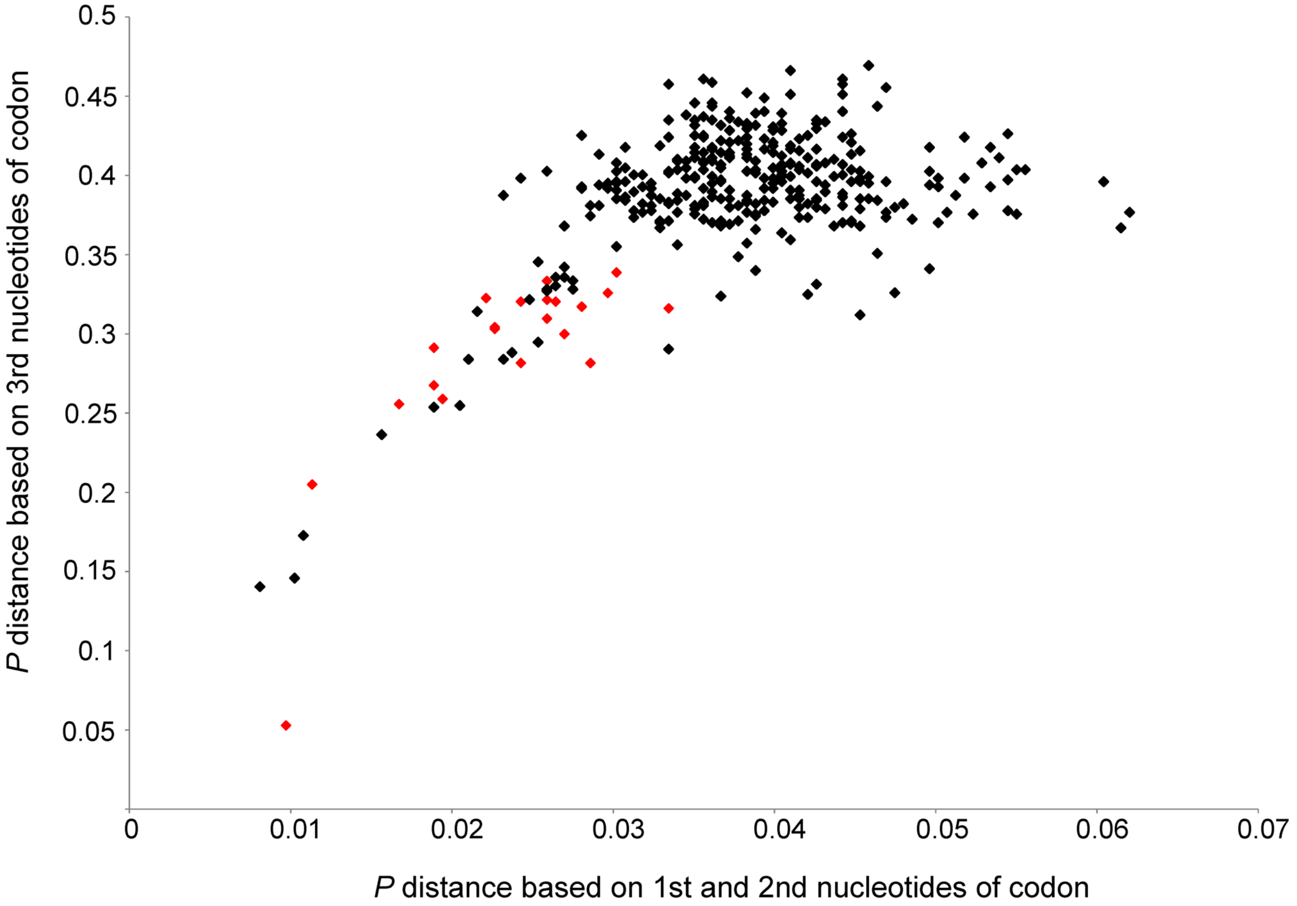

Figure G. Comparison of *p* distances among the liagoracean species based on the first and second nucleotides of codons and based on the third nucleotide of codons, in the combined *psaA* and *rbc*L dataset used for the present phylogenetic analyses (Fig. 1). Red diamonds indicate *p* distances among "*Liagora*" *japonica* and four related genera: *Akalaphycus*, *Macrocarpus*, *Neoizziella*, and *Titanophycus*.
