# Supplementary material for: Next-Generation Sequencing of an 88-Year-Old Specimen of the Poorly Known Species Liagora japonica (Nemaliales, Rhodophyta) Supports the Recognition of Otohimella gen. nov
Source: PLoS One. 2016 Jul 7;11(7):e0158944. doi: 10.1371/journal.pone.0158944 (PMC4936710; doi:10.1371/journal.pone.0158944)
Supplement: S2 File — Table A. Collection and herbarium information for specimens of Liagora japonica used in the morphological analyses that have no molecular data. Table B. Collection locations and details, and GenBank accession numbers of samples used in the psaA, psaB, psbA, rbcL, COI, 18S rRNA, and 28S rRNA genes analyses. Table C. GenBank accession numbers of species used in the identification of Liagora japonica sequences from old herbarium specimens. Table D. Target read coverage of Liagora japonica (sample ID: suzuki-2; sample No.: OJ1). Table E. Target read coverage of Liagora japonica (sample ID: suzuki-1; sample No.: OJ2). Table F. De novo assembly statistics. Table G. Summary for the Bayesian analyses on the basis of psaA, psaB, psbA, rbcL, 18S rRNA, and 28S rRNA datasets. Table H. Matrix of p distances of the liagoracean species used in the COI analysis. Table I. Matrix of Kimura 2-parameter (K2P) genetic distances of the liagoracean species used in the COI analysis. (DOC) [file pone.0158944.s002.doc]

**Table A. Collection and herbarium information for specimens of *Liagora japonica*** used in the morphological analyses that have no molecular data.

| Sample No. | Collection data |
| --- | --- |
| OJ5 | Misaki (35°15' N, 139°36' E), Miura, Kanagawa Prefecture, Japan; May 1903; TI; lectotype |
| OJ6 | Oura (34°40' N, 138°56' E), Shimoda, Shizuoka Prefecture, Japan; 15 May 1952; coll. Mitsuo Chihara; TNS-AL 047542 |
| OJ7 | Kisami (34°39' N, 138°55' E), Shimoda, Shizuoka Prefecture, Japan; 26 Mar. 1959; TNS-AL 029956 |
| OJ8 | Hamashima (34°17' N, 136°45' E), Mie Prefecture, Japan; 1 Apr. 1938; coll. Michitaro Higashi; TNS-AL 158161 |
| OJ9 | Kushimoto (33°26' N, 135°45' E), Higashimuro County, Wakayama Prefecture, Japan; 29 Apr. 1960; coll. Torao Yamamoto; TNS-AL 150505 |
| OJ10 | Tsutsu (34°06' N, 129°10' E), Tsushima, Nagasaki Prefecture, Japan; 17 Mar. 1969; coll. Mitsuo Chihara & Makoto Yoshizaki; TNS-AL 029955^a^ |
| OJ11 | Nishiura (34°06' N, 129°10' E), Tsutsu, Tsushima, Nagasaki Prefecture, Japan; 15 Mar. 1969; coll. Mitsuo Chihara & Makoto Yoshizaki; TNS-AL 029954 |
| OJ12 | Uragawa (38°12' N, 138°29' E), Sado, Niigata Prefecture, Japan; 21 Feb. 2003; coll. Masahiro Suzuki; TNS-AL 185627 |
| OJ13 | Uragawa (38°12' N, 138°29' E), Sado, Niigata Prefecture, Japan; 7 Nov. 2003; coll. Masahiro Suzuki; TNS-AL 185628 |
| OJ14 | Tamasaki (38°09' N, 138°27' E), Sado, Niigata Prefecture, Japan; 26 Jul. 1998; coll. Makoto Yoshizaki; TNS-AL 190026 |
| OJ15 | Sai (41°27' N, 140°52' E), Shimokita County, Aomori Prefecture, Japan; 25 Oct. 1987; coll. Taiju Kitayama; SAP 53016^b^ |

^a^Voucher specimen of Chihara and Yoshizaki (1972).

^b^165 bp of *rbc*L sequence is identical to LC066217.

**Table B. Collection locations and details, and GenBank accession numbers of samples used in the plastid-encoded *psaA*, *psaB, psbA, rbc*L, the universal mitochondrial barcode marker COI, and nuclear-encoded 18S *r*RNA and 28S *r*RNA gene** analyses.

| Sample No. | Collection data | *psaA* | *psaB* | *psbA* | *rbc*L | COI | 18S *r*RNA | 28S *r*RNA |
| --- | --- | --- | --- | --- | --- | --- | --- | --- |
| OJ1 | Misaki (35°15' N, 139°36' E), Miura, Kanagawa Prefecture, Japan; May 1927; coll. Yukio Yamada; SAP88755; topotype | LC066222 | LC093495 | LC093497 | LC066217 | LC066521 | LC066529 | LC066525 |
| OJ2 | Nada (33°49' N, 135°10' E), Gobou, Wakayama Prefecture. Japan; 20 Apr. 1958; coll. Torao Yamamoto; SAP 28242 | LC066223 | LC093496 | LC093498 | LC066218 | LC066522 | LC066530 | LC066526 |
| OJ3 | Uragawa (38°12' N, 138°29' E), Sado, Niigata Prefecture, Japan; 6 Aug. 2011; coll. Masahiro Suzuki; TNS-AL 182118 |  |  |  | LC066219 | LC066523 | LC066531 | LC066527 |
| OJ4 | Jodogaura (36°18' N, 133°20' E), Okinoshimacho, Oki County, Shimane Prefecture, Japan; 26 Jul. 2014; coll. Akira Kurihara; TNS-AL 195934 |  |  |  | LC066220 | LC066524 | LC066532 | LC066528 |

**Table C. GenBank accession numbers of species used in the identification of *Liagora japonica* sequences from historical herbarium specimens.**

| Species | Reference | *psaA* | *psaB* | *psbA* | *rbc*L | COI | 28S *r*RNA | 18S *r*RNA |
| --- | --- | --- | --- | --- | --- | --- | --- | --- |
| Nemaliales |  |  |  |  |  |  |  |  |
| *Akalaphycus liagoroides* (Yamada) Huisman, I.A. Abbott & A.R. Sherwood | [1] |  |  |  | KC134343 |  |  | KC157584 |
| *Akalaphycus setchelliae* (Yamada) Huisman, I.A. Abbott & A.R. Sherwood | [2] |  |  |  | GU357697 |  |  |  |
| *Ganonema farinosum* (J.V. Lamouroux) K.C. Fan & Yung C. Wang | [3] |  |  |  | KF667109 |  |  |  |
| *Gloiocallis dendroidea* (P. Crouan & H. Crouan) Showe M. Lin, Huisman & D.L. Ballantine (as *Ganonema dendroideum*) | [3] |  |  |  | KF667101 |  |  |  |
| *Hommersandiophycus borowitzkae* (Huisman) Showe M. Lin & Huisman | [3] |  |  |  | KF667093 |  |  |  |
| *Hommersandiophycus clavatus* (Yamada) Showe M. Lin & Huisman | [3] |  |  |  | KF667098 |  |  |  |

**Table C. Continued.**

| Species | Reference | *psaA* | *psaB* | *psbA* | *rbc*L | COI | 28S *r*RNA | 18S *r*RNA |
| --- | --- | --- | --- | --- | --- | --- | --- | --- |
| *Hommersandiophycus pectinatus* (Collins & Hervey) Popolizio, C.W. Schneider & C.E. Lane |  |  |  |  |  | HQ603226 |  |  |
| *Hommersandiophycus samaensis* (C.K. Tseng) Showe M. Lin & Huisman | [3] |  |  |  | KF667102 |  |  |  |
| *Izziella formosana* (Yamada) Showe M. Lin, S.-Y. Yang & Huisman | [2] |  |  |  | GU357688 |  |  |  |
| *Izziella hommersandii* Showe M. Lin, S.-Y. Yang & Huisman | [2] |  |  |  | GU357690 |  |  |  |
| *Izziella kuroshioensis* Showe M. Lin, S.-Y. Yang & Huisman | [2] |  |  |  | GU357684 |  |  |  |
| *Izziella orientalis* (J. Agardh) Huisman & Schils | [4] |  |  |  |  | HQ422594 |  |  |
| *Liagora albicans* J.V. Lamouroux | [5] |  |  |  | HQ901786 |  |  |  |

**Table C.** Continued.

| Species | Reference | *psaA* | *psaB* | *psbA* | *rbc*L | COI | 28S *r*RNA | 18S *r*RNA |
| --- | --- | --- | --- | --- | --- | --- | --- | --- |
| *Liagora albicans* J.V. Lamouroux | [4] |  |  |  |  | HQ422860 |  |  |
|  | [4] |  |  |  |  | HQ422866 |  |  |
|  | [4] |  |  |  |  | HQ422978 |  |  |
| *Liagora boergesenii* Yamada | [2] |  |  |  | GU357679^a^ |  |  |  |
|  | [4] |  |  |  |  | HQ422649 |  |  |
| *Liagora ceranoides* J.V. Lamouroux | [2] |  |  |  | GU357681 |  | GU357669 |  |
| *Liagora distenta* (Mertens ex Roth) J.V. Lamouroux | [6] |  |  |  |  | HQ603225 |  |  |
| *Liagora donaldiana* I.A. Abbott & Huisman | [4] |  |  |  |  | HQ423078 |  |  |
| *Liagora harveyana* Zeh | [2] |  |  |  | HM572263 |  |  |  |
|  | [7] |  |  |  |  | DQ873275 |  |  |
| *Liagora japonica* Yamada (OJ3) | This study |  |  |  | LC066219 | LC066523 | LC066527 | LC066531 |
| *Liagora japonica* Yamada (OJ4) | This study |  |  |  | LC066220 | LC066524 | LC066528 | LC066532 |
| *Liagora julieae* I.A. Abbott & Huisman | [4] |  |  |  | HQ422852 |  |  |  |

**Table C.** Continued.

| Species | Reference | *psaA* | *psaB* | *psbA* | *rbc*L | COI | 28S *r*RNA | 18S *r*RNA |
| --- | --- | --- | --- | --- | --- | --- | --- | --- |
| *Liagora mannarensis* V. Krishnamurthy & Sundararajan | [8] |  |  |  |  |  | AY570374 |  |
| *Liagora viscida* (Forsskål) C. Agardh | [2] |  |  |  | GU357678 |  | GU357670 |  |
|  | [6] |  |  |  |  | HQ603227 |  |  |
| *Liagora* sp. | [4] |  |  |  |  | HQ422634 |  |  |
|  | [4] |  |  |  |  | HQ422780 |  |  |
|  | [4] |  |  |  |  | HQ422954 |  |  |
|  | [4] |  |  |  |  | HQ422956 |  |  |
| *Macrocarpus perennis* (I.A. Abbott) Showe M. Lin, S.-Y. Yang & Huisman (as *L*. *perennis*) | [5] |  |  |  | HQ901783 |  |  |  |
| *Neoizziella asiatica* Showe M. Lin, S.-Y. Yang & Huisman | [5] |  |  |  | HQ901777 |  |  |  |
| *Neoizziella divaricata* (C.K. Tseng) Showe M. Lin, S.-Y. Yang & Huisman | [5] |  |  |  | HQ901781 |  |  |  |

**Table C. Continued.**

| Species | Reference | *psaA* | *psaB* | *psbA* | *rbc*L | COI | 28S *r*RNA | 18S *r*RNA |
| --- | --- | --- | --- | --- | --- | --- | --- | --- |
| *Neoizziella divaricata* (C.K. Tseng) Showe M. Lin, S.-Y. Yang & Huisman | [4] |  |  |  |  | HQ423117 |  |  |
| *Nemalion multifidum* (Lyngbye) Chauvin (as *Nemalion* sp.) | Unpublished | DQ787598 |  |  |  |  |  |  |
| *Nemalion multifidum* (Lyngbye) Chauvin | [1] |  |  |  |  |  |  | KC157579 |
| *Nothogenia fastigiata* (Bory) P.G. Parkinson | [1] |  |  |  |  |  |  | KC157581 |
| *Stenopeltis gracilis* (Yamada & Tak. Tanaka) Itono & Yoshizaki | [2] |  |  |  | GU357695 |  |  |  |
| *Titanophycus setchellii* (Yamada) Showe M. Lin, S.-Y. Yang & Huisman (as *L*. *setchelii*) | [2] |  |  |  | GU357694 |  | GU357674 |  |
| *Titanophycus validus* (Harvey) Huisman, G.W. Saunders & A.R. Sherwood | [2] |  |  |  | GU357692 |  | GU357672 |  |
| *Trichogloeopsis mucosissima* (Yamada) I.A. Abbott & Doty | [3] |  |  |  | KF667107 |  |  |  |

**Table C. Continued.**

| Species | Reference | *psaA* | *psaB* | *psbA* | *rbc*L | COI | 28S *r*RNA | 18S *r*RNA |
| --- | --- | --- | --- | --- | --- | --- | --- | --- |
| *Trichogloeopsis pedicellata* (M. Howe) I.A. Abbott & Doty | [3] |  |  |  | KF667108 |  |  |  |
| *Yoshizakia indopacifica* Showe M. Lin, Huisman & C. Payri | [9] |  |  |  | JX878374 |  |  |  |
| Nemaliophycidae |  |  |  |  |  |  |  |  |
| *Acrochaetium savianum* (Meneghini) Nägeli | Unpublished | DQ787597 |  |  |  |  |  |  |
| *Ballia callitriche* (C. Agardh) Kützing | Unpublished | DQ787595 |  |  |  |  |  |  |
| *Batrachospermum gelatinosum* (Linnaeus) De Candolle | Unpublished | DQ787596 |  |  |  |  |  |  |
| *Palmaria palmata* (Linnaeus) F. Weber & D. Mohr | Unpublished | DQ787599 |  |  |  |  |  |  |
| *Thorea violacea* Bory | [10] | AY119712 |  |  |  |  |  |  |

**Table C. Continued.**

| Species | Reference | *psaA* | *psaB* | *psbA* | *rbc*L | COI | 28S *r*RNA | 18S *r*RNA |
| --- | --- | --- | --- | --- | --- | --- | --- | --- |
| Corallinophycidae |  |  |  |  |  |  |  |  |
| *Calliarthron tuberculosum* (Postels & Ruprecht) E.Y. Dawson | [11] |  | KC153978^b^ | KC153978^b^ |  |  |  |  |
| Rhodymeniophycidae |  |  |  |  |  |  |  |  |
| *Chondrus crispus* Stackhouse | [11] |  | HF562234^b^ | HF562234^b^ |  |  |  |  |
| *Gracilaria Salicornia* (C. Agardh) E.Y. Dawson | [12] |  | KF861575^b^ | KF861575^b^ |  |  |  |  |
| *Grateloupia taiwanensis* Showe M. Lin & H.Y. Liang | [13] |  | KC894740^b^ | KC894740^b^ |  |  |  |  |

^a^The sequence is identical with *Dotyophycus yamadae* (Ohmi & Itono) I.A. Abbott & Yoshizaki (JX878366).

^b^Complete genome of plastid.

**Table D. Target read coverage of *Liagora japonica* (sample ID: suzuki-2; sample No.: OJ1).**

|  | *psaA* | *psaB* | *psbA* | *rbc*L | COI | 18S *r*RNA | 28S *r*RNA |
| --- | --- | --- | --- | --- | --- | --- | --- |
| Reconstructed gene sequence length (bp) | 2259 | 2205 | 1083 | 1467 | 1599 | 2675 | 1619 |
| Average read coverage per base | 538.8 | 438.1 | 577.3 | 673.9 | 321.3 | 198 | 217.5 |
| (Total length of mapped reads)/2 (bp) | 1217746 | 966283 | 625515.5 | 988827 | 514100 | 529928 | 352419.5 |
| Average mapped read length (bp) | 180.5 | 181.8 | 180.4 | 183.7 | 184.2 | 165.9 | 170.5 |
| Standard deviation of mapped read length (bp) | 69 | 67.8 | 68.8 | 69.6 | 68.9 | 68.9 | 67.7 |
| Total number of mapped pairs to the reference gene | 6744 | 5314 | 3466 | 5382 | 2790 | 3194 | 2066 |
| Number of mapped identical sequence pairs between F and R read | 4382 | 3379 | 2252 | 3569 | 1852 | 1806 | 1493 |
| Number of mapped different sequence pairs between F and R read | 2362 | 1935 | 1214 | 1813 | 938 | 1388 | 573 |
| Number of mapped different sequence pairs with only F or R read was mapped to the reference gene | 422 | 397 | 168 | 148 | 102 | 136 | 35 |
| Percent of identical sequence pairs in total mapped pairs to the reference gene | 64.9 | 63.5 | 64.9 | 66.3 | 66.3 | 56.5 | 72.2 |

**Table E. Target read coverage of *Liagora japonica* (sample ID: suzuki-1; sample No.: OJ2).**

|  | *psaA* | *psaB* | *psbA* | *rbc*L | COI | 18S *r*RNA | 28S *r*RNA |
| --- | --- | --- | --- | --- | --- | --- | --- |
| Reconstructed gene sequence length (bp) | 2259 | 2205 | 1083 | 1467 | 1599 | 2675 | 1619 |
| Average read coverage per base | 76.9 | 60.5 | 86 | 101.1 | 128.4 | 27.5 | 25.2 |
| (Total length of mapped reads)/2 (bp) | 173875 | 133476 | 93274.5 | 148404 | 205554 | 73802.5 | 40874.5 |
| Average mapped read length (bp) | 146.7 | 149.3 | 141.9 | 147 | 153.1 | 137.9 | 138 |
| Standard deviation of mapped read length (bp) | 57.5 | 57.9 | 57 | 56.2 | 62.3 | 54.4 | 48.6 |
| Total number of mapped pairs to the reference gene | 1185 | 894 | 657 | 1009 | 1342 | 535 | 296 |
| Number of mapped identical sequence pairs between F and R read | 975 | 735 | 533 | 841 | 1101 | 390 | 259 |
| Number of mapped different sequence pairs between F and R read | 210 | 159 | 124 | 168 | 241 | 145 | 37 |
| Number of mapped different sequence pairs with only F or R read was mapped to the reference gene | 25 | 19 | 20 | 7 | 25 | 19 | 2 |
| Percent of identical sequence pairs in total mapped pairs to the reference gene | 82.2 | 82.2 | 81.1 | 83.3 | 82 | 72.8 | 87.5 |

**Table F. De novo assembly statistics.**

| Sample ID (Sample No.) | Number of high quality reads (pairs) | Number of >500 bp scaffolds | Largest scaffolds (bp) | N50 length (bp) |
| --- | --- | --- | --- | --- |
| suzuki-1 (OJ2) | 10,057,049 | 101,376 | 45,375 | 619 |
| suzuki-2 (OJ1) | 8,913,992 | 158,568 | 32,912 | 670 |

**Table G. Summary for the Bayesian analyses on the basis of *psaA*, *psaB*, *psbA*, *rbc*L, COI, 18S *r*RNA, and 28S *r*RNA datasets.**

|  | *psaA* + *rbc*L | *psaA* | *rbc*L | COI | 28S *r*RNA | 18S *r*RNA | *psaB* | *psbA* |
| --- | --- | --- | --- | --- | --- | --- | --- | --- |
| Number of taxa | 45 | 49 (55)^*1^ | 72 (99)^*1^ | 22 (21) ^*1^ | 49 (51)^*1^ | 19 (21)^*1^ | 8 (9)^*1^ | 16 |
| Number of nucleotides (bp) included in analysis | *psaA*: 1407, *rbc*L: 1374 | 1392 | 1317 | 579 | 2082^*2^ | 1453^*2^ | 2205 | 834 |
| Substitution model selected^*3^ | *psaA*: 1st codons (GTR+I+G), 2nd codons (GTR+I+G), 3rd codons (GTR+I+G)  *rbc*L: 1st codons (GTR+I+G), 2nd codons (F81+I+G), 3rd codons (GTR+I+G) | 1st codons (GTR+I+G), 2nd codons (GTR +I+G), 3rd codons (GTR+I+G) | 1st codons (GTR+I+G), 2nd codons (F81+I+G), 3rd codons (GTR+I+G) | 1st codons (GTR+G), 2nd codons (F81), 3rd codons (GTR+G) | GTR+I+G | GTR+I+G | 1st codons (GTR+G), 2nd codons (GTR+I+G), 3rd codons (GTR+G) | 1st codons (SYM+G), 2nd codons (JC), 3rd codons (GTR+G) |

**Table G. Continued.**

|  | *psaA* + *rbc*L | *psaA* | *rbc*L | COI | 28S *r*RNA | 18S *r*RNA | *psaB* | *psbA* |
| --- | --- | --- | --- | --- | --- | --- | --- | --- |
| MCMC generations | 2,000,000 | 2,000,000 | 3,000,000 | 2,000,000 | 2,000,000 | 2,000,000 | 2,000,000 | 2,000,000 |
| Average standard deviation of split | 0.003956 | 0.006071 | 0.009248 | 0.002545 | 0.003587 | 0.008603 | 0.001131 | 0.001975 |

^*1^ The numbers within parentheses indicate original number of taxa including the samples with identical nucleotide sequences.

^*2^Numbers of aligned sites.

^*3^Each substitution model was selected by hierarchical likelihood ratio test using MrModeltest 2.3 [14].

**Table H. Matrix of *p* distances among the liagoracean species used in the COI analysis.**

|  | 1 | 2 | 3 | 4 | 5 | 6 | 7 | 8 | 9 |
| --- | --- | --- | --- | --- | --- | --- | --- | --- | --- |
| 1. *Liagora japonica* OJ1 | - |  |  |  |  |  |  |  |  |
| 2. *Liagora* *japonica* OJ2 | 0.00518135 | - |  |  |  |  |  |  |  |
| 3. *Liagora* *japonica* OJ3, OJ4 | 0.01554404 | 0.01381693 | - |  |  |  |  |  |  |
| 4. *Neoizziella* *divaricata* HQ423117 | 0.13644214 | 0.13816926 | 0.13816926 | - |  |  |  |  |  |
| 5. *Liagora donaldiana* HQ423078 | 0.12953368 | 0.13298792 | 0.13126080 | 0.12953368 | - |  |  |  |  |
| 6. *Liagora* *boergesenii* HQ422649 | 0.15889464 | 0.15716752 | 0.15716752 | 0.14335060 | 0.13471502 | - |  |  |  |
| 7. *Liagora* sp. HQ422634 | 0.15544042 | 0.15371330 | 0.15371330 | 0.14853196 | 0.13298792 | 0.01381693 | - |  |  |
| 8. *Izziella_orientalis* HQ422594 | 0.16234888 | 0.16407600 | 0.16062176 | 0.17616580 | 0.14335060 | 0.17962003 | 0.17098446 | - |  |
| 9. *Liagora albicans* HQ422978 | 0.15544042 | 0.15025906 | 0.14853196 | 0.15889464 | 0.14680484 | 0.18307427 | 0.17271157 | 0.12435233 | - |
| 10. *Liagora albicans* HQ422866 | 0.14853196 | 0.14335060 | 0.14162348 | 0.16062176 | 0.14680484 | 0.18652850 | 0.17616580 | 0.12607944 | 0.01899827 |
| 11. *Liagora albicans* HQ422860 | 0.16234888 | 0.16062176 | 0.16407600 | 0.16062176 | 0.16407600 | 0.18134715 | 0.17443869 | 0.13989638 | 0.07772021 |
| 12. *Liagora distenta* HQ603225 | 0.15198618 | 0.15198618 | 0.15371330 | 0.16925734 | 0.16580310 | 0.18652850 | 0.17962003 | 0.12435233 | 0.11917099 |
| 13. *Liagora julieae* HQ422852 | 0.15556994 | 0.15556994 | 0.15037246 | 0.15385078 | 0.15396003 | 0.18666779 | 0.17975710 | 0.12443700 | 0.11410315 |
| 14. *Liagora viscida* HQ603227 | 0.16407600 | 0.16925734 | 0.16234888 | 0.16580310 | 0.16753022 | 0.18307427 | 0.17616580 | 0.12089810 | 0.11226252 |
| 15. *Liagora* sp. HQ422956 | 0.16925734 | 0.16925734 | 0.17271157 | 0.15716752 | 0.16753022 | 0.18998273 | 0.18652850 | 0.13989638 | 0.12435233 |
| 16. *Liagora* sp. HQ422954 | 0.15544042 | 0.15544042 | 0.15716752 | 0.16234888 | 0.15198618 | 0.16234888 | 0.15544042 | 0.13816926 | 0.12607944 |
| 17. *Liagora* sp. HQ422780 | 0.15371330 | 0.15371330 | 0.15025906 | 0.16925734 | 0.16062176 | 0.16580310 | 0.16234888 | 0.14680484 | 0.12953368 |
| 18. *Hommersandiophycus pectinatus* HQ603226 | 0.16753022 | 0.16234888 | 0.16580310 | 0.17271157 | 0.16234888 | 0.17616580 | 0.17443869 | 0.18825561 | 0.16062176 |

**Table H. Continued.**

|  | 10 | 11 | 12 | 13 | 14 | 15 | 16 | 17 | 18 |
| --- | --- | --- | --- | --- | --- | --- | --- | --- | --- |
| 1. *Liagora japonica* OJ1 |  |  |  |  |  |  |  |  |  |
| 2. *Liagora* *japonica* OJ2 |  |  |  |  |  |  |  |  |  |
| 3. *Liagora* *japonica* OJ3, OJ4 |  |  |  |  |  |  |  |  |  |
| 4. *Neoizziella* *divaricata* HQ423117 |  |  |  |  |  |  |  |  |  |
| 5. *Liagora donaldiana* HQ423078 |  |  |  |  |  |  |  |  |  |
| 6. *Liagora* *boergesenii* HQ422649 |  |  |  |  |  |  |  |  |  |
| 7. *Liagora* sp. HQ422634 |  |  |  |  |  |  |  |  |  |
| 8. *Izziella_orientalis* HQ422594 |  |  |  |  |  |  |  |  |  |
| 9. *Liagora albicans* HQ422978 |  |  |  |  |  |  |  |  |  |
| 10. *Liagora albicans* HQ422866 | - |  |  |  |  |  |  |  |  |
| 11. *Liagora albicans* HQ422860 | 0.07944732 | - |  |  |  |  |  |  |  |
| 12. *Liagora distenta* HQ603225 | 0.11917099 | 0.13298792 | - |  |  |  |  |  |  |
| 13. *Liagora julieae* HQ422852 | 0.11409407 | 0.11581545 | 0.13137032 | - |  |  |  |  |  |
| 14. *Liagora viscida* HQ603227 | 0.11053541 | 0.13126080 | 0.11571676 | 0.12101270 | - |  |  |  |  |
| 15. *Liagora* sp. HQ422956 | 0.12262522 | 0.11398964 | 0.13298792 | 0.13138475 | 0.12435233 | - |  |  |  |
| 16. *Liagora* sp. HQ422954 | 0.12607944 | 0.12780656 | 0.11226252 | 0.12790917 | 0.13989638 | 0.11917099 | - |  |  |
| 17. *Liagora* sp. HQ422780 | 0.12780656 | 0.13471502 | 0.13298792 | 0.12274799 | 0.11917099 | 0.13471502 | 0.12953368 | - |  |
| 18. *Hommersandiophycus pectinatus* HQ603226 | 0.16234888 | 0.16753022 | 0.17789292 | 0.19011766 | 0.19516407 | 0.17443869 | 0.16753022 | 0.18134715 | - |

**Table I. Matrix of Kimura 2-parameter (K2P) genetic distances among the liagoracean species used in the COI analysis.**

|  | 1 | 2 | 3 | 4 | 5 | 6 | 7 | 8 | 9 |
| --- | --- | --- | --- | --- | --- | --- | --- | --- | --- |
| 1. *Liagora japonica* OJ1 | - |  |  |  |  |  |  |  |  |
| 2. *Liagora* *japonica* OJ2 | 0.00520838 | - |  |  |  |  |  |  |  |
| 3. *Liagora* *japonica* OJ3, OJ4 | 0.01576537 | 0.01398917 | - |  |  |  |  |  |  |
| 4. *Neoizziella* *divaricata* HQ423117 | 0.15241556 | 0.15464273 | 0.15448944 | - |  |  |  |  |  |
| 5. *Liagora donaldiana* HQ423078 | 0.14292258 | 0.14725161 | 0.14498559 | 0.14283040 | - |  |  |  |  |
| 6. *Liagora* *boergesenii* HQ422649 | 0.18217263 | 0.17981967 | 0.17960888 | 0.16009565 | 0.15005483 | - |  |  |  |
| 7. *Liagora* sp. HQ422634 | 0.17747775 | 0.17514674 | 0.17494684 | 0.16679217 | 0.14785218 | 0.01401142 | - |  |  |
| 8. *Izziella_orientalis* HQ422594 | 0.18406512 | 0.18636397 | 0.18189923 | 0.20175615 | 0.15938573 | 0.20703724 | 0.19524424 | - |  |
| 9. *Liagora albicans* HQ422978 | 0.17557563 | 0.16878819 | 0.16643274 | 0.17974012 | 0.16420557 | 0.21166740 | 0.19744845 | 0.13709708 | - |
| 10. *Liagora albicans* HQ422866 | 0.16654603 | 0.15987927 | 0.15758295 | 0.18202844 | 0.16420557 | 0.21649836 | 0.20214331 | 0.13925691 | 0.01936862 |
| 11. *Liagora albicans* HQ422860 | 0.18446855 | 0.18216440 | 0.18663678 | 0.18202844 | 0.18611851 | 0.20897038 | 0.19953614 | 0.15721501 | 0.08336194 |
| 12. *Liagora distenta* HQ603225 | 0.17104045 | 0.17104045 | 0.17343797 | 0.19362989 | 0.18818893 | 0.18818893 | 0.20688269 | 0.13709708 | 0.13126765 |
| 13. *Liagora julieae* HQ422852 | 0.17561246 | 0.17561246 | 0.16893576 | 0.17323226 | 0.17277952 | 0.21591265 | 0.20640452 | 0.13668761 | 0.12446852 |
| 14. *Liagora viscida* HQ603227 | 0.18600610 | 0.19291864 | 0.18383019 | 0.18818893 | 0.19072685 | 0.21107565 | 0.20164141 | 0.13303448 | 0.12273866 |
| 15. *Liagora* sp. HQ422956 | 0.19318236 | 0.19318236 | 0.19801950 | 0.17746221 | 0.19072685 | 0.21967866 | 0.21493928 | 0.15687987 | 0.13776125 |
| 16. *Liagora* sp. HQ422954 | 0.17557563 | 0.17557563 | 0.17771989 | 0.1843273 | 0.17031585 | 0.18461655 | 0.17544200 | 0.15464273 | 0.13913736 |
| 17. *Liagora* sp. HQ422780 | 0.17343797 | 0.17343797 | 0.16878819 | 0.19347382 | 0.18189923 | 0.18854196 | 0.18394423 | 0.16455002 | 0.14418115 |
| 18. *Hommersandiophycus pectinatus* HQ603226 | 0.18993880 | 0.18316668 | 0.18760966 | 0.19657850 | 0.18311524 | 0.20259441 | 0.20022474 | 0.21760963 | 0.18104354 |

**Table I. Continued.**

|  | 10 | 11 | 12 | 13 | 14 | 15 | 16 | 17 | 18 |
| --- | --- | --- | --- | --- | --- | --- | --- | --- | --- |
| 1. *Liagora japonica* OJ1 |  |  |  |  |  |  |  |  |  |
| 2. *Liagora* *japonica* OJ2 |  |  |  |  |  |  |  |  |  |
| 3. *Liagora* *japonica* OJ3, OJ4 |  |  |  |  |  |  |  |  |  |
| 4. *Neoizziella* *divaricata* HQ423117 |  |  |  |  |  |  |  |  |  |
| 5. *Liagora donaldiana* HQ423078 |  |  |  |  |  |  |  |  |  |
| 6. *Liagora* *boergesenii* HQ422649 |  |  |  |  |  |  |  |  |  |
| 7. *Liagora* sp. HQ422634 |  |  |  |  |  |  |  |  |  |
| 8. *Izziella_orientalis* HQ422594 |  |  |  |  |  |  |  |  |  |
| 9. *Liagora albicans* HQ422978 |  |  |  |  |  |  |  |  |  |
| 10. *Liagora albicans* HQ422866 | - |  |  |  |  |  |  |  |  |
| 11. *Liagora albicans* HQ422860 | 0.08537807 | - |  |  |  |  |  |  |  |
| 12. *Liagora distenta* HQ603225 | 0.13126765 | 0.14877491 | - |  |  |  |  |  |  |
| 13. *Liagora julieae* HQ422852 | 0.12445746 | 0.12637727 | 0.14652897 | - |  |  |  |  |  |
| 14. *Liagora viscida* HQ603227 | 0.12062895 | 0.14655268 | 0.12674767 | 0.13370769 | - |  |  |  |  |
| 15. *Liagora* sp. HQ422956 | 0.13558733 | 0.12538333 | 0.14877491 | 0.14654747 | 0.13776125 | - |  |  |  |
| 16. *Liagora* sp. HQ422954 | 0.13913736 | 0.14130187 | 0.12353490 | 0.14109367 | 0.15672159 | 0.13038744 | - |  |  |
| 17. *Liagora* sp. HQ422780 | 0.14198335 | 0.15050350 | 0.14894989 | 0.13574156 | 0.13113694 | 0.15050350 | 0.14335290 | - |  |
| 18. *Hommersandiophycus pectinatus* HQ603226 | 0.18329073 | 0.19027661 | 0.20341456 | 0.21977170 | 0.22666119 | 0.19880623 | 0.18993880 | 0.20798585 | - |

**Additional References**

1. Scott FJ, Saunders GW, Kraft GT. *Entwisleia bella*, gen. et sp. nov., a novel marine ‘batrachospermaceous’ red alga from southeastern Tasmania representing a new family and order in the Nemaliophycidae. Euro. J. Phycol. 2013; 48: 398–410.

2. Lin S-M, Yang S-Y, Huisman JM. Systematic revision of the genus *Liagora* and *Izziella* (Liagoraceae, Rhodophyta) from Taiwan based on molecular analyses and carposporophyte development, with the description of two new species. J. Phycol. 2011; 47: 352–365.

3. Lin S-M, Huisman JM, Ballantine DL. Revisiting the systematics of *Ganonema* (Liagoraceae, Rhodophyta) with emphasis on species from the northwest Pacific Ocean. Phycologia 2014; 53: 37–51.

4. Sherwood AR, Kurihara A, Conklin KY, Sauvage T, Presting GG. The Hawaiian Rhodophyta Biodiversity Survey (2006-2010): a summary of principal findings. BMC Plant Biol. 2010: 10: 258.

5. Lin S-M, Yang S-Y, Huisman JM. Systematics of *Liagora* with diffuse gonimoblasts based on *rbc*L sequences and carposporophyte development, including the description of the new genera *Neoizziella* and *Macrocarpus* (Liagoraceae, Rhodophyta). Euro. J. Phycol. 2011; 46: 249–262.

6. Le Gall L, Saunders GW. Establishment of a DNA-barcode library for the Nemaliales (Rhodophyta) from Canada and France uncovers overlooked diversity in the species *Nemalion helminthoides* (Velley) Batters. Cryptogamie Algol. 2010; 31: 403-421.

7. Huisman JM, Saunders GM, Sherwood AR. Recognition of *Titanophycus*, a new genus based on *Liagora valida* Harv. (Liagoraceae, Nemaliales). In: Huisman JM, editors. Algae of Australia – Nemaliales: ABRS; 2006. pp. 116-119.

8. Huisman JM, Harper JT, Saunders GW. Phylogenetic study of the Nemaliales (Rhodophyta) based on large-subunit ribosomal DNA sequences supports segregation of the Scinaiaceae fam. nov. and resurrection of *Dichotomaria* Lamarck. Phycol Res 2004; 52: 224–234.

9. Lin S-M, Huisman JM, Payli C. Characterization of *Liagora ceranoides* (Liagoraceae, Rhodophyta) on the basis of *rbc*L sequence analyses and carposporophyte development, including *Yoshizakia indopacifica* gen. et sp. nov. from the Indo-Pacific region. Phycologia 2013; 52: 161–170.

10. Yoon HS, Hackett JD, Bhattacharya D. A single origin of peridinin- and fucoxanthin-containing plastids in dinoflagellates through tertiary endosymbiosis. PNAS 2002; 99: 11724-11729.

11. Janouskovec J, Liu SL, Martone PT, Carre W, Leblanc, C, et al. Evolution of red algal plastid genomes: ancient architectures, introns, horizontal gene transfer, and taxonomic utility of plastid markers. PLOS ONE 2013; 8: e59001.

12. Campbell MA, Presting GP, Bennett MS, SherwoodAR. Highly conserved organellar genomes in the Gracilariales as inferred using new data from the Hawaiian invasive alga *Gracilaria salicornia*. Phycologia 2014; 53: 109-116.

13. Depriest MS, Bhattacharya D, Lopez-Bautista JM. The Plastid Genome of the Red Macroalga *Grateloupia taiwanensis* (Halymeniaceae). PLOS ONE 2013; 8: e 68246.

14. Nylander JAA. MrModeltest 2.1. Program distributed by the author. Evolutionary Biology Centre, Uppsala University, Uppsala; 2004.
