# Supplementary material for: Next-Generation Sequencing of an 88-Year-Old Specimen of the Poorly Known Species Liagora japonica (Nemaliales, Rhodophyta) Supports the Recognition of Otohimella gen. nov
Source: PLoS One. 2016 Jul 7;11(7):e0158944. doi: 10.1371/journal.pone.0158944 (PMC4936710; doi:10.1371/journal.pone.0158944)
Supplement: S1 Text — (DOCX) [file pone.0158944.s003.docx]

**S1 Text. The DNA extraction and sequencing procedures for modern specimens.**

The modern specimens used in molecular analyses are listed in S1 Table. Total DNA was extracted from field-collected specimens dried by silica gel using the DNeasy Plant Mini Kit (QIAGEN, Tokyo, Japan) following the instructions of the manufacturer. The total DNA was used as a template for polymerase chain reaction (PCR) amplification using a TOYOBO KOD FX Neo (TOYOBO CO. LTD., Oosaka, Japan). Primers used for PCR amplification were: 28S *r*RNA gene: T01 (5’-TAAGCATATCAGTAAGCGGAG-3’) – V (5’-CGTATCGCCAGTTCTGCTTACC-3’), F449 (5’-CCCGAAGATGGTGAACTATG-3’) – G (5’-CACCACGTCCTCCTACTC-3’), T04 (5’-GCAGGACGGTGGCCATGGAAGT-3’) - 28F (5’-CAGAGCACTGGGCAGAAAATCAC-3’), and T05 (5’-GCAACGGKCAAAGGGAATCCG-3’) - T15 (5’-TGATAGGAAGAGCCGACATCGA-3’) [1, 2]; 18S rRNA gene: SR1 (5’-CCTGGTTGATCCTGCCAG-3’) - SR9 (5’-AACTAAGAACGGCCATGCAC-3’), and SR4 (5’-AGCCGCGGTAATTCCAGCT-3’) - SR12 (5’-CCTTCYGCAGGTTCACCTAC-3’) [3]; *rbc*L: F8 (5’-GGYGTAATTCCATATGCWAAAATG-3’) - R1150 (5’-GCATTTGWCCACARTGAATACC-3’) and F645 (5’-ATGMGHTGGAAAGAAAGATT-3’) - R1381 (5’- ATCTTTCCATAAATCTARAGC-3’) [4]; COI: GazF1 (5’-TCAACAAATCATAAAGATATTGG-3’) - GazR1 (5’-ACTTCTGGATGTCCAAAAAAYCA-3’) [5]. The temperature-cycling protocol was: 28S *r*RNA gene: 2 min at 94°C for an initial denaturation step, followed by 35 cycles of 15 sec denaturation at 94°C, 30 sec primer annealing at 55°C, and 1 min extension at 68°C, with a final 7 min extension at 72°C, and then a hold at 4°C; *rbc*L and COI: 2 min at 94°C for an initial denaturation step, followed by 35 cycles of 15 sec denaturation at 94°C, 30 sec primer annealing at 46°C and 1 min extension at 68°C, with a final 7 min extension at 72°C, and then a hold at 4°C. The amplified DNA fragments were purified using QIAquick PCR Purification Kit (QIAGEN, Tokyo, Japan). Cycle-sequencing with BigDye Terminator v3.1 Cycle Sequencing Kit (Applied Biosystems, Tokyo, Japan) was carried out in a 7.5 μl volume of reaction: 2 μl of BigDye Terminator v3.1 Reaction Mix, 10-30 ng/ml of PCR product, 10 pmol of primer, and dH_2_O to 7.5 μl. The cycle-sequencing program consisted of an initial step at 97°C for 2 min, 25 sequencing cycles (97°C for 10 s, 50°C for 25 s, 60°C for 2 min). The BigDye-labeled PCR products were ethanol-precipitated following the manufacturer's protocol and completely sequenced using ABI PRISM 310 Genetic Analyzer (Applied Biosystems, Tokyo, Japan). Reverse and direct chromatograms were assembled using the program GeneStudio^TM^ Professional Ver. 2.2. (GeneStudio, Inc.).

**Additional References**

1. Freshwater DW, Fredericq S, Bailey JC. Characteristics and utility of nuclear-encoded large-subunit ribosomal gene sequences in phylogenetic studies of red algae. Phycol Res 1999; 47: 33–38.

2. Harper JT, Saunders GW. The application of sequences of the ribosomal cistron to the systematics and classification of the florideophyte red algae (Florideophyceae, Rhodophyta). Cah Biol Mar 2001; 42: 25–38.

3. Nakayama T, Watanabe S, Mitsui K, Uchida H, Inouye I. The phylogenetic relationship between the Chlamydomonadales and Chlorococcales inferred from 18SrDNA sequence data. Phycol Res 1996; 44: 47-55.

4. Wang HW, Kawaguchi S, Horiguchi T, Masuda M. Reinstatement of *Grateloupia catenata* (Rhodophyta, Halymeniaceae) on the basis of morphology and *rbc*L sequences. Phycologia 2000; 39: 228–237.

5. Saunders GW. Applying DNA barcoding to red macroalgae: a preliminary appraisal holds promise for future applications. Phil Trans R Soc B 2005; 360: 1879-1888.
